# Spatial microenvironments tune immune response dynamics in the *Drosophila* larval fat body

**DOI:** 10.1101/2024.09.12.612587

**Published:** 2024-09-16

**Authors:** Brandon H. Schlomann, Ting-Wei Pai, Jazmin Sandhu, Genesis Ferrer Imbert, Thomas G.W. Graham, Hernan G. Garcia

**Affiliations:** 1 Department of Molecular and Cell Biology, University of California, Berkeley, CA, USA; 2 Department of Physics, University of California, Berkeley, CA, USA; 3 Institute for Quantitative Biosciences-QB3, University of California, Berkeley, CA, USA; 4 Chan Zuckerberg Biohub – San Francisco, San Francisco, CA, USA; 5 Biophysics Graduate Group, University of California, Berkeley, CA, USA; 6 Graduate Program in Bioengineering, University of California, Berkeley, CA, USA

## Abstract

Immune responses in tissues display intricate patterns of gene expression that vary across space and time. While such patterns have been increasingly linked to disease outcomes, the mechanisms that generate them and the logic behind them remain poorly understood. As a tractable model of spatial immune responses, we investigated heterogeneous expression of antimicrobial peptides in the larval fly fat body, an organ functionally analogous to the liver. To capture the dynamics of immune response across the full tissue at single-cell resolution, we established live light sheet fluorescence microscopy of whole larvae. We discovered that expression of antimicrobial peptides occurs in a reproducible spatial pattern, with enhanced expression in the anterior and posterior lobes of the fat body. This pattern correlates with microbial localization via blood flow but is not caused by it: loss of heartbeat suppresses microbial transport but leaves the expression pattern unchanged. This result suggests that regions of the tissue most likely to encounter microbes via blood flow are primed to produce antimicrobials. Spatial transcriptomics revealed that these immune microenvironments are defined by genes spanning multiple biological processes, including lipid-binding proteins that regulate host cell death by the immune system. In sum, the larval fly fat body exhibits spatial compartmentalization of immune activity that resembles the strategic positioning of immune cells in mammals, such as in the liver, gut, and lymph nodes. This finding suggests that tissues may share a conserved spatial organization that optimizes immune responses for antimicrobial efficacy while preventing excessive self-damage.

## Introduction

Immune responses in tissues exhibit complex spatiotemporal patterns of gene expression and cellular behaviors [1–4]. Recent advances in our ability to map immune responses in space at single-cell resolution have led to increased identification of gene expression patterns in immune microenvironments that correlate with disease severity in infections [5, 6] and cancer [7, 8]. A fundamental open problem in immunology is understanding the various mechanisms that generate these patterns and the logic behind them.

Mechanisms for generating spatial patterns of immune responses include processes that are cell-autonomous and those that arise from cell-cell or cell-environment interactions. Within individual cells, transcription is a stochastic process that generates strong heterogeneity in mRNA levels [9–13], and genes involved in innate immunity are known to be even more variable in their expression than the typical gene [14]. Further, positive feedback loops and amplification steps in immune signaling pathways can create complex dynamics of gene expression that can increase variability [15]. In terms of cell-cell interactions, communication via secreted cytokines can generate spatial patterns of immune activity with tunable length scales [16]. In terms of cell-environment interactions, the anatomical structure of tissues can have a large influence on the spatial structure of cellular activities. For example, in lymph nodes, macrophages strategically occupy the sinus lining of the node to rapidly detect microbial signals and activate lymphoid cells more interior to the node, resulting in cytokine signaling patterns that mirror the anatomy of the tissue [1]. More generally, the spatial distribution and behavior of microbes can be a strong driver of immune activity patterns [17, 18]. Understanding how all of these processes acting at different length and time scales combine to generate observed spatial patterns of immune response remains an open problem.

A significant challenge in deciphering spatial patterns of immune response is the lack of dynamic information, particularly at the single-cell level, which limits our ability to understand how spatial patterns emerge and evolve. Given the multiscale nature of the problem, being able to follow the dynamics of gene expression in individual cells across a whole tissue would provide direct insight into how processes at different scales interact with one another. For example, it would clarify whether the stochastic dynamics of individual cells are relevant or negligible when measured against the spatial variation that arises from the anatomical structure of tissues.

Currently, measurements of single-cell gene expression dynamics and tissue-scale spatial patterns are generally done in separate experiments. Single-cell gene expression dynamics are typically confined to in vitro cell culture settings, where the spatial and physiological complexities of the tissue environment are removed. Such experiments have revealed deep insights into the complex dynamics that can arise from cell signaling and transcription [19–22], but how these dynamics interact with the tissue environment is often unknown. In contrast, recent advances in sequencing and imaging technologies enable snapshots of the full complexity of tissue immune responses at exquisite scale and resolution [2, 3, 5, 6]. Yet, the dynamics that generate these snapshots cannot be directly observed but must be inferred [23], limiting our ability to identify mechanisms that drive cellular variability.

To address this barrier, we established live imaging of immune responses in fruit fly larvae, a well-established, optically transparent model of innate immunity. Using light sheet fluorescence microscopy [24, 25], fluorescent reporters of gene expression [26], and computational image analysis, we demonstrated the ability to quantitatively measure immune response dynamics across the whole animal (~3mm long) with single-cell resolution during systemic infections. We used this approach to investigate previous reports [27, 28] of heterogeneous expression of antimicrobial peptides within an organ called the fat body, which serves similar functions to those of the mammalian liver and adipose tissue combined [29].

By capturing the full, intact tissue, we unexpectedly discovered that, while expression of antimicrobial peptides appears locally random, it exhibits a robust global spatial pattern, with enhanced expression in the anterior and posterior regions of the tissue. Within mosaic regions, cell-cell variability is driven by a continuum of single-cell production rates, rather than all-or-nothing activation. This global spatial pattern of immune response correlates with, but is not caused by, microbial localization via blood flow. Thus, our data, along with a reanalysis of published spatial transcriptomes [30], indicate the presence of persistent immune microenvironments within the larval fat body that are primed to respond to systemic infections.

Together, our findings suggest that the anterior and posterior fat body are primed for immune response because of their proximity to regions of elevated microbial exposure via blood flow. This situation resembles the concentration of leukocytes at the portal vein of the mammalian liver [31], as well as other examples of strategic cellular positioning at interfaces with high microbial exposure such as the gut [32] and lymph nodes [1]. Based on these observations, we propose a conserved spatial logic of tissues, where the anatomy and physical structure of organs shape spatiotemporal patterns of immune response to optimize antimicrobial efficacy while minimizing unnecessary activation.

In summary, this work contributes a significant improvement in the ability to quantitatively measure the dynamics of gene expression patterns at single-cell resolution during in vivo immune responses. The combination of these live imaging methods and the powerful genetic toolkit of the fruit fly enables the dissection of how tissue immune response dynamics are regulated at the single-cell level. Our discovery of spatial microenvironments within the larval fat body could significantly enhance our understanding of the broader physiology of the fly, particularly in terms of the integration of its circulatory and immune systems [33]. More generally, this finding supports the notion of strategic cellular positioning [1] within immunological tissues as a conserved design principle of the immune system.

## Results

### DptA and other antimicrobial peptides are expressed in a robust spatial pattern within the fat body

Fruit flies possess a highly conserved innate immune system, in which microbial ligands are sensed by pattern recognition receptors that activate a range of antimicrobial functions via signaling through NF-*κ*B-type transcription factors [29]. A core component of the fly immune response is the production of antimicrobial peptides [29], various combinations of which are required for surviving different microbes [34]. During systemic infections, large amounts of antimicrobial peptides are secreted from an organ called the fat body, which is functionally similar to a combination of the mammalian liver and adipose tissue [29].

One antimicrobial peptide that exhibits spatial heterogeneity in its expression is Diptericin-A (DptA) [27, 28, 35]. Expression from a DptA-LacZ reporter exhibits a mosaic response across the fat body in a manner dependent on the steroid hormone Ecdysone [27, 28, 35–38]. Larvae late in the developmental stage known as third instar have high levels of Ecdysone and thus strong DptA expression in response to infection on average. In contrast, larvae in early third instar and earlier have lower Ecdysone levels and thus exhibit a reduced DptA response on average, which manifests as mosaic expression patterns. Thus, by precisely staging larvae at different points within the third instar stage, one can tune the degree of heterogeneous DptA expression. We sought to use the mosaic expression of DptA as a model system for identifying mechanisms driving cell-cell variability in immune responses. We measured DptA expression using an established GFP-based transcriptional reporter [26], referred to here as DptA-GFP.

To study mechanisms driving cell-cell variability in DptA expression within the fat body, we developed a protocol based on precise larval staging and microinjection-based infection (Fig. S1A–B) that produces DptA-GFP responses that are heterogeneous yet reproducible, with 100% of larvae contained some amount of detectable DptA-GFP signal (Fig. S1C). We checked that DptA-GFP levels do indeed represent a linear, quantitative measure of gene expression by measuring that animals homozygous for the reporter contain on a median fluorescence intensity approximately twice that of heterozygous animals (Fig. S2A; medians and their bootstrapped standard deviations: 7 ± 4 · 10^4^ a.u., homozygotes, 3.2 ± 0.6 · 10^4^ a.u., heterozygotes). We also confirmed that the ether anesthetic used to immobilize larvae for injections does not affect DptA-GFP levels by comparing to larvae immoblized by cold shock (Fig. S2B).

**Figure 1:**
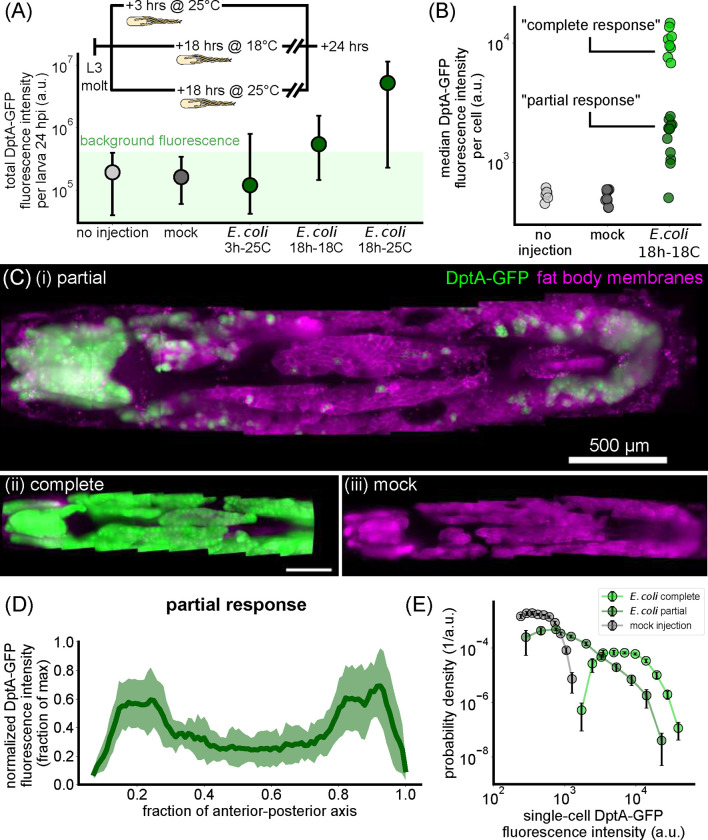
The antimicrobial peptide reporter DptA-GFP is expressed heterogeneously throughout the fat body but exhibits a reproducible spatial pattern along the anterior-posterior axis during early third instar. (A) The inducibility of DptA increases with larval age. Total fluorescence intensity of DptA-GFP per larva at 24 hours after infection with *E. coli* is plotted as a function of age after molt to L3. Inset shows the experimental timeline. Circles denote median values, bars denote quartiles. Age is denoted by hours after molt to L3 at a given temperature in degrees Celsius. Larvae aged 18 hours post L3 molt at 18°C at the time of infection produce intermediate DptA expression levels, and are the focal age of the paper. No injection and mock groups showed no detectable DptA-GFP signal and thus represent the measured range of background fluorescence. (B) From image-based quantification of single-cell DptA-GFP levels, we plot the median single-cell expression level for each larva and find that larvae cluster into two groups, denoted “partial responses” and “complete responses”. (C) Maximum intensity projections of larvae showing DptA-GFP (green) and fat body membranes (magenta, r4-Gal4 x UAS-mCD8-mCherry). A representative partial response (i) exhibits high expression in the anterior- and posterior-dorsal fat body, with minimal, scattered expression in the middle fat body. Complete responses (ii) exhibit a uniform expression pattern, while mock injected larvae (iii) show no detectable expression. Timing is 24 hours after injection. DptA-GFP channel is log-transformed and all images are adjusted to the same contrast levels. Scale bar in (ii) is 500 *μ*m. (D) Quantification of the “U-shaped” DptA-GFP expression pattern for partial responses only. Each larva’s expression pattern is normalized to its maximum value and then averaged (green line). Shaded error bars denote standard deviation across N=12 larvae. (E) Probability densities of single-cell DptA-GFP expression levels for mock (gray), partial responses (dark green), and complete responses (bright green), showing that partial responses comprise a continuous, broad distribution of expression levels.

With our infection protocol established, we began by measuring total DptA-GFP fluorescence intensity 24 hours after infection, long after the initial activation of DptA, which occurs between 3–5 hours post infection [27]. Due to the high stability of GFP in vivo [39], this measurement is a proxy for the total amount of Diptericin produced over the course of the infection. Both mock injected and non-injected larvae produced zero observable DptA-GFP signal (Fig. 1A, gray circles). Comparing larvae injected at different times within early third instar (3 hours post molt to third instar at 25°C, 18 hours at 18°C, and 18 hours at 25°C), we observed a monotonic increase of DptA-GFP levels with developmental stage (Fig. 1A, green circles). We found that injecting larvae at the 18h-18C time point produced responses with the strongest within-fat body heterogeneity and chose to characterize this stage further.

Using image analysis (Methods), we quantified DptA-GFP fluorescence intensities within individual cells. Larvae from this stage cleanly clustered into two populations based on median cell intensity (Fig. 1B), which we denote as “complete response” and “partial response”. These two clusters did not correlate with fat body length, which is a proxy for developmental stage and thus Ecdysone levels, or experiment date (Fig. S3A). We did observe a partial correlation with larva sex, with 6/6 male larvae exhibiting a partial response, which might be indicative of X-linked Ecdysone effects [27], though female larvae were split evenly across partial and complete responses (6 partial, 8 complete). Since larvae of both sexes exhibited examples of partial responses, we continued to analyze both males and females in all experiments. Complete responses were uniform across the fat body (Fig. 1C,ii). In contrast, partial responses were highly heterogeneous (Fig. 1C,i, Supplemental Movie 1); mock-injected larvae showed no detectable expression (Fig. 1C,iii).

Remarkably, cellular variability in DptA expression had a highly stereotyped spatial pattern: mean expression was high in the anterior and posterior fat body, but lower in the middle (Fig. 1D, plot shows mean and standard deviation across N=12 larvae; expression profiles along the anterior-posterior axis were normalized to the maximum level to account for variability in the overall level of DptA expression). The balance of expression between anterior and posterior fat body varied between larvae: some larvae had stronger expression in the anterior than in the posterior, some had the reverse, but one or both of anterior and posterior always had between 2 and 10 times higher mean expression than the middle (Fig. S3B). This spatial pattern of DptA expression was independent of injection site on the larva (Fig. S2C–F). Quantitative inspection of these responses at the single-cell level revealed that partial responses exhibit a broad, continuous distribution of expression levels that ranges from zero detectable expression (consistent with mock injections) all the way up to levels consistent with complete responses (Fig. 1E, Fig. S3C).

This robust spatial pattern of DptA expression was a surprise to us, as previous work, which used LacZ reporters containing the same 2.2kb DptA regulatory sequence as our GFP reporter [26], found the response to be a random, “salt-and-pepper” pattern [27]. We suspect the discrepancy is due to our high bacterial load (~10^5^ bacteria per larva), which reduces stochasticity in the response, and possibly to our ability to measure the response across the full, intact fat body tissue, which is difficult to do by staining dissected tissue fragments.

**Figure 2:**
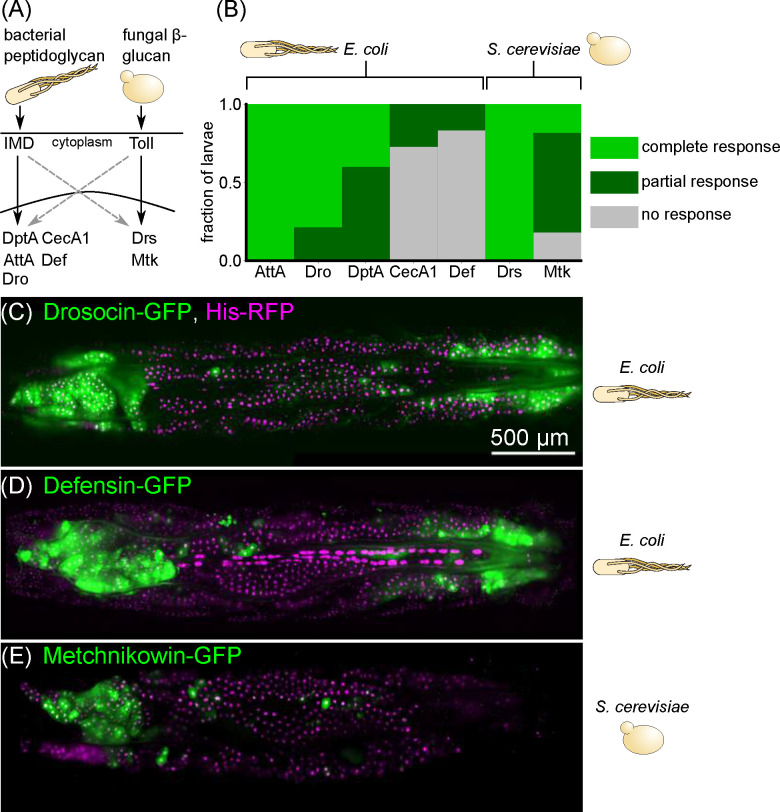
Spatial patterns of expression upon immune challenge occur in a variety of antimicrobial peptides. (A) Highly simplified schematic of the main immune signaling pathways in *Drosophila*. Bacterial peptidoglycan is sensed through the immune deficient (IMD) pathway, which leads to activation of Diptericins (including DptA), Cecropins (including CecA1), Attacins (including AttA), Defensin (Def), and Drosocin (Dro). Fungal *β*-glucan is sensed through the Toll pathway and leads to activation of Drosomycin (Drs) and Metchnikowin (Mtk). There is cross-talk between the pathways (dashed gray arrows). (B) Fraction of larvae exhibiting partial (subset of fat body cells GFP^+^), complete (all fat body cells GFP^+^), or no response of GFP-reporters of various antimicrobial peptides following challenge with *E. coli* or *S. cerevisiae*. Responses were scored based on images taken on a low-magnification widefield microscope 24 hours post infection, except for DptA, which were taken from the light sheet fluorescence microscopy data from Fig. 1. All larvae were staged to 18h post-L3 molt at 18°C (Methods). Sample sizes (number of larvae) for each gene, left to right: N=7,14,20,11,12,8,11. (C)-(E) Maximum intensity projections of light sheet fluorescence microscopy image stacks of larvae carrying GFP reporters for Drosocin (C), Defensin (D), and Metchnikowin (E), with the microbial stimulus used noted to the right of each image. Fat body nuclei are marked using r4-Gal4 X UAS-HisRFP. Image contrast was adjusted for each panel separately for visual clarity.

We next examined whether this spatial expression pattern was unique to DptA, or was shared among other antimicrobial peptides. We screened a suite of 6 additional reporter constructs [26] that span the full family of classical antimicrobial peptides in fruit flies: Attacin, Cecropin, Defensin, Drosocin, Drosomycin, and Metchnikowin. Specifically, we asked whether larvae with partial responses, in which only a subset of fat body cells express the reporter, exhibited a similar “U-shaped” expression pattern to DptA. The first 4 antimicrobial peptides are known to be downstream of the IMD pathway in the fat body [40] (Fig. 2A) and so were induced using the same *E. coli* infection protocol as DptA. The last two peptides are known to be induced in the fat body primarily by the Toll pathway [40], which senses Lys-type peptidoglycan and fungal *β*-glucan (Fig. 2A), and so were induced by microinjection with yeast, *S. cerevisiae* (Methods). We found that antimicrobial peptide responses varied considerably both within and across peptides (Fig. 2B). Attacin-A and Drosomycin were strongly expressed in all fat body cells in all larvae, and so we were unable to assess the spatial patterning of partial responses for these genes. In contrast, Cecropin-A1 was barely detectable, with only a small number of cells in a small number of larvae positive for GFP, preventing robust assessment of spatial patterning of this gene. However, Drosocin and Defensin exhibited clear examples of a “U-shaped” partial response, mirroring DptA (Fig. 2C–D). Metchnikowin exhibited strong expression only in the anterior, not posterior fat body (Fig. 2E). Together, these data indicate that spatial patterning of immune response in the larval fat body—particularly enhanced expression in the anterior-dorsal lobes—is not restricted to DptA, but is a more general phenomenon that spans IMD and Toll pathways.

With this repeatable yet heterogeneous immune response expression pattern characterized, we next sought to understand the origins of both cell-cell variability within fat body regions and the overall spatial patterning across the tissue. We began by leveraging our live imaging capabilities to characterize the dynamics of immune response pattern formation.

### Single-cell DptA-GFP expression dynamics are deterministic with spatially-varying rates

Variability in DptA-GFP levels 24 hours after infection could arise from multiple different types of dynamics. The highest expressing cells could have the highest rates of DptA expression, the shortest delay before beginning to respond, the largest fluctuations as part of a highly stochastic response, or a combination thereof. To distinguish between these dynamical modes of activation, we adapted our light sheet fluorescence microscopy mounting protocol to enable continuous imaging of live larvae for several hours (Methods) and obtained movies of two larvae exhibiting partial responses.

**Figure 3:**
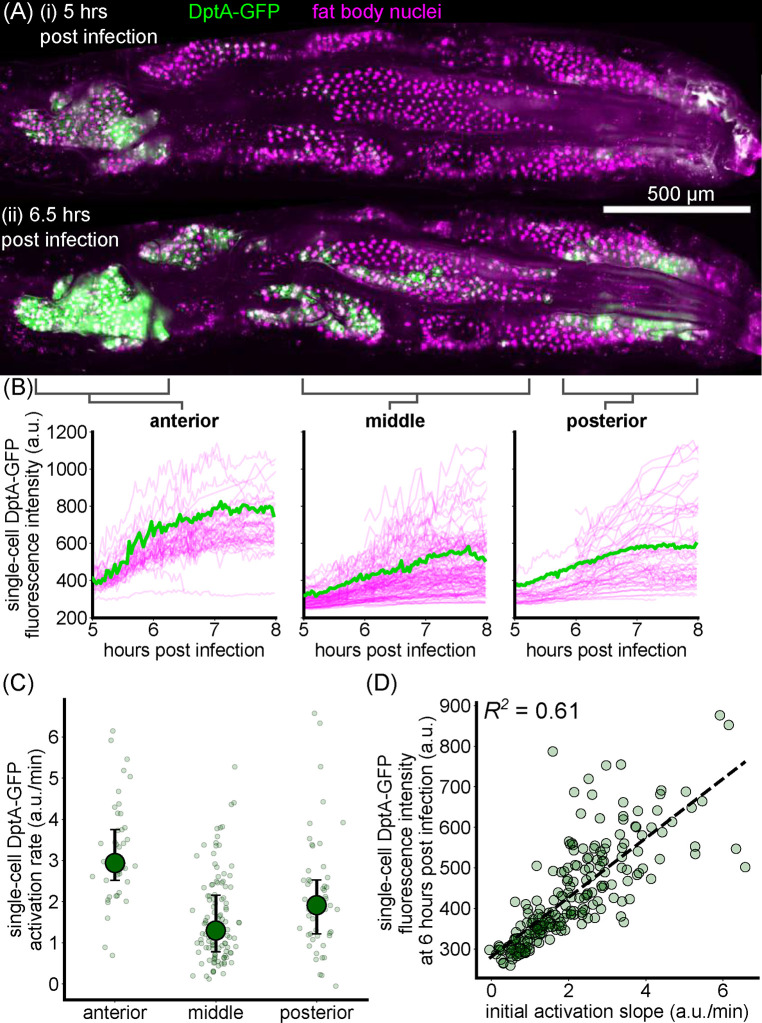
Single-cell dynamics of DptA expression exhibit smooth activation with spatially-varying rates. (A) Maximum intensity projection snapshots of DptA-GFP expression during time-lapse imaging. Time denotes hours post infection. The images come from Supplemental Movie 2. See also Supplemental Movie 3. (B) Single-cell traces of mean DptA-GFP expression per cell over time from cells in 3 regions of the dorsal fat body. One representative trace from each region is highlighted in green, the rest are drawn in magenta. The data are pooled from movies of N=2 larvae (Supplemental Movies 2 and 3). (C) Single-cell DptA-GFP activation rates in anterior, middle, and posterior regions of the fat body. Large circles and error bars denote quartiles. Small circles represent individual cells. (D) Instantaneous fluorescence intensity 6 hours post infection strongly correlates with the initial rate of production. Each marker is a cell.

Levels of DptA-GFP visibly increased over the course of the movies, with a clear bias of expression in the anterior-dorsal lobes of the fat body (Fig. 3A, Supplemental Movies 2 and 3). Using image analysis (Methods), we quantified the dynamics of expression in 227 cells across 2 movies and pooled the data for analysis (Fig. 3B). While each single-cell measurement contained substantial noise due to fluctuating background levels and tissue motion, the overall trends were smooth increases in DptA levels in all cells, with spatially varying rates. Fitting a linear rise to the initial phase of activation, we found that single-cell activation rates in the anterior fat body are uniformly high compared to rest of the tissue, with a median rate roughly twice that of the middle region (Fig. 3C). The middle region contains more variability, with a continuous spread of rates ranging from zero expression all the way to rates consistent with the anterior (Fig. 3B–C). The posterior region has a 60% higher median rate than the middle, though there is also a wide, continuous spread in the rates. The posterior region also showed the most variability between the two larvae we analyzed, which is evident in the movies (Supplemental Movies 2 and 3), and also has the highest level of autofluorescent background due to the gut, which makes analysis less accurate (see the posterior region of Fig. 3A,i). Finally, there is a moderate correlation ($R^{2}\;=\;0.61$) between the initial activation rate and the level of DptA expression at 6 hours post infection (Fig. 3D), suggesting that expression rate, not delay, primarily determines long-term expression level. Due to the high stability of GFP, our measurements are insensitive to potential high frequency fluctuations in DptA expression. However, overall, the data support a model of largely deterministic expression with spatially varying rates, rather than one of varying activation delays or strongly stochastic dynamics.

We validated our light sheet fluorescence microscopy-based measurements by manually following individual larvae for several hours on a widefield microscope (Methods, Fig. S4). While this approach does not allow segmentation of single cells due to strong background fluorescence, we were able to quantify tissue-scale activation dynamics in the anterior fat body. We observed a similar pattern of smooth increase in expression that resulted in signal that is approximately twice as bright as background levels by 6 hours post infection.

Having inferred that DptA patterning is due primarily to a deterministic modulation of expression rate, we searched for the drivers of this variability, beginning with the bacteria themselves.

### Bacterial transport through the heart correlates with, but does not cause, DptA patterning

**Figure 4:**
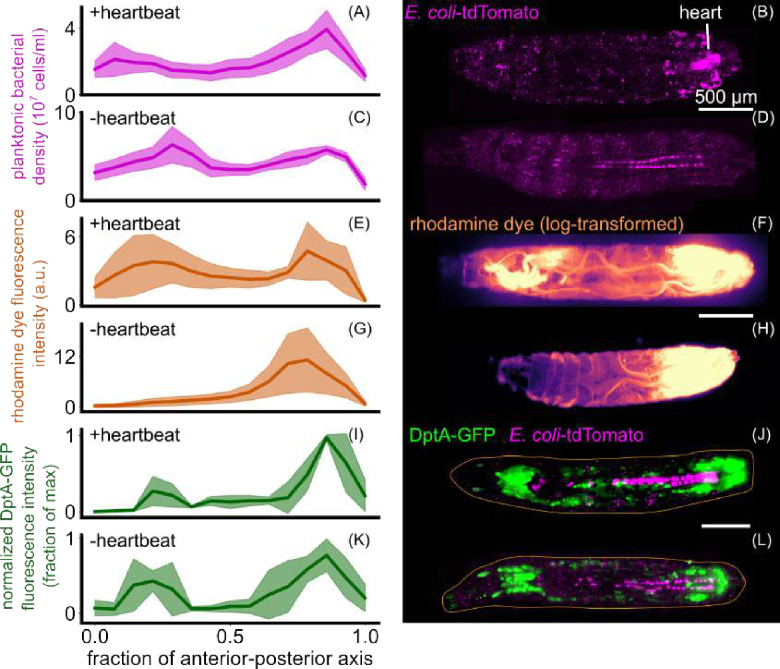
Heartbeat-induced fluid flows pattern bacteria and dye but are not required for patterning of DptA. Each row shows quantification (left, mean and standard deviation) and a representative image (right) of various quantities. (A)-(D) *E. coli* 3 hours post injection with and without a heartbeat (N=4 larvae per group). In the quantification, to avoid counting fluorescence internalized by host cells, planktonic bacteria freely suspended in the hemolymph were computationally identified and only these cells were counted (Methods). The heartbeat was eliminated by myosin knockdown in the heart using NP1029-Gal4 x UAS-Mhc-RNAi. (E)-(H) Rhodamine dye injected in the posterior and imaged 5 minutes after injection, with and without a heartbeat (N=5 larvae per group). (I)-(L) DptA-GFP 6 hours post injection in animals with and without a heartbeat (N=5 larvae per group). All scale bars are 500 *μ*m. In (J) and (L), the approximate outline of the larva is marked as an orange line. Images in (B), (D) (J), and (L) are maximum intensity projections of 3D light sheet images stacks. Images in (F) and (H) are single-plane widefield images.

DptA is activated by the Imd pathway, which in turn is activated by the binding of peptidoglycan to a membrane-bound receptor [29]. Therefore, we hypothesized that the observed spatial pattern of DptA expression might be caused by spatial localization of bacteria. The insect hemolymph is generally thought of as a well-mixed environment due to the open circulatory system, but recently has been recognized to be capable of spatial compartmentalization and other complex flows [41]. To test the hypothesis of bacteria localization, we injected larvae with fluorescent *E. coli*-tdTomato [42] and imaged 3–5 hours post injection, right before the peak of DptA expression [27]. Bacteria were present throughout the animal (Fig. 4A–B). Large numbers of planktonic bacteria were observed suspendend in the hemolymph (Fig. S5A). We also detected a consistent concentration of bacterial signal in the posterior that appeared to correspond to the heart (Fig. 4B, S5B). In addition, we observed clusters of bacteria preferentially localized in bands along the larvae, suggesting that they were internalized by phagocytic hemocytes that reside in band patterns known as sessile clusters (Fig. S5C) [43]. Finally, the larval heart contains cells called nephrocytes that absorb and filter contents of the hemolymph [44, 45]. We observed fluorescent signal within nephrocyte-like cells along the heart, which could be due to true bacteria or to the internalization of excess tdTomato protein released by bacterial cells into the hemolymph (Fig. S5D).

Using computational image analysis, we segmented individual bacteria and bacterial aggregates and normalized all objects to the median single-cell intensity, resulting in a quantitative map of bacterial cell counts and aggregation behavior (Methods, Supplemental Movie 5). Normalizing by larval volume, we obtained a measurement of bacterial cell density along the anterior-posterior axis. As indicated in the images, we measured a strong peak in bacterial density in the posterior due to aggregation on the heart, though density throughout the rest of the animal is uniform (Fig. S6A). We suspected that the bulk of the signal was coming from bacterial aggregates, some of which appeared to reside within phagocytic cells that are uniformly distributed along the body wall. As the extent to which phagocytosed bacteria contribute to antimicrobial peptide activation is unclear, we computationally extracted only the bacterial density that corresponded to planktonic bacteria, which are more likely to be suspended in the hemolymph (Methods). Restricting to only planktonic cells, we retained a strong posterior peak in bacterial density and gained a small peak in the anterior (Fig. 4A), resulting in a pattern that qualitatively resembles DptA expression. However, plotting DptA-GFP fluorescence intensity against planktonic bacterial density in the same anterior-posterior axis bins revealed distinct input-output relationships in the anterior and posterior regions, suggesting that average bacterial concentration is not the sole determinant of DptA expression (Fig. S6B).

To further characterize the distribution of bacteria in the hemolymph, we took movies of a single optical plane of the light sheet. We found that the hemolymph is a highly dynamic fluid environment, and observed that bacteria are directly transported through the heart (technically known as the “dorsal vessel”), flowing from the posterior to the anterior at a speed of approximately 1 mm/s (Supplemental Movie 6). These observations of bacterial transport through the heart, along with the fact that the anterior opening of the heart exists close to the anterior-dorsal lobes of the fat body ([46]), led us to hypothesize that blood flow, rather than average bacterial localization per se, was required for spatially-patterned DptA expression. We envisioned two non-exclusive mechanisms by which blood flow would lead to spatial patterning of antimicrobial peptides: first, by facilitating increased binding of bacterial peptidoglycan to fat body membrane-bound receptors; and second, by pro-immune signaling via mechanotransduction, as was recently shown for hemocyte differentiation in the lymph glands [47]. To test role of blood flow on DptA patterning, we genetically eliminated the heartbeat.

The larval circulatory system consists of a single tube suspended in the hemolymph that pumps peristaltically from the posterior to the anterior at a frequency of around 4 Hz [46]. The heartbeat can be controlled by genetic perturbations using the larval heart-specific Gal4 driver NP1029 [46, 47]. We eliminated the heartbeat by knocking down myosin heavy chain (Mhc) specifically in the heart using NP1029-Gal4 driving UAS-Mhc-RNAi (Supplemental Movie 7), following reference [47]. In animals lacking a heartbeat, the average distribution of planktonic bacteria along the anterior-posterior axis shifted only slightly towards the posterior (Fig. 4C–D). We also note that nephrocyte-localized signal still occurred in the absence a heartbeat (Fig. 4D). To directly measure fluid transport in the hemolymph, we injected larvae with rhodamine dye and imaged them 5 minutes post injection. Phenotypically wild-type larvae containing only NP1029-Gal4 showed rapid (<10 seconds) transport of dye from posterior injection site to the anterior that remained visible at 5 minutes post injection (Fig. 4E–F). The location of the anterior pool of dye overlapped with the location of the high DptA-expressing anterior-dorsal lobes of the fat body (Fig. 4E). As expected, loss of heartbeat completely eliminated dye transport through the heart (Fig. 4H–G). The flow of bacteria through the heart was also eliminated, though additional fluid flows were still present due to body wall contractions (Supplemental Movie 8).

We then combined our DptA-GFP reporter with the heart-specific myosin knockdown and assessed DptA levels 6 hours post infection (recall that DptA levels at this time point correlate with activation rates at the single cell level; Methods; Fig. S7). Control larvae containing only the reporter and UAS-Mhc-RNAi showed the expected “U-shaped” expression pattern along the anterior-posterior axis (Fig. 4I,J). In contradiction to our hypothesis, larvae lacking a heartbeat also showed a strong “U-shaped” expression pattern, indicating that the heartbeat is not required for spatially-patterned DptA expression (Fig. 4K,L).

In addition to knocking down myosin, we eliminated the heartbeat by overexpressing the potassium channel Ork1, following [46]. While this strategy robustly eliminated the heartbeat throughout the larval stage, unexpectedly, we found that after injection with either *E. coli* or a mock control, the heartbeat restarted within 3–6 hours (Supplemental Movie 9), preventing us from using this approach to assess the role of the heartbeat in DptA expression. We note that the spatial pattern of DptA expression was unchanged by Ork1 overexpression (Fig. S8).

Altogether, these results establish that the observed spatial patterning of antimicrobial peptides within the fat body correlates with, but is not caused by, bacterial transport via blood flow. Therefore, we inferred that these regions of enhanced immune activity in the fat body represent persistent spatial microenvironments that are primed for antimicrobial peptide expression prior to the start of infection. Since our heartbeat knockdown was in effect from the beginning of embryogenesis, we could conclude that the heartbeat itself is not involved in the immune priming. We next searched for factors that define these microenvironments at baseline.

### Spatial transcriptomics reveals spatially patterned genes within the unperturbed fat body, including the host-protective factor Turandot-A.

Given that Ecdysone signaling leads to stronger DptA expression on average [27], we first asked if the observed spatial pattern in DptA expression could be explained by a spatial pattern of Ecdysone Receptor (EcR) nuclear localization. Ecdysone is secreted in its precursor form in pulses throughout the larval stage from the prothoracic gland [36], which is located in the anterior of the larva, near the anterior-dorsal lobes of the fat body that exhibit strong DptA expression. Therefore, we hypothesized that DptA expression in the anterior fat body might be explained by temporary spatial gradients in Ecdysone signaling. To test this hypothesis, we used a recently made fly line containing an endogenously-tagged B1 subunit of Ecdysone Receptor, mNeonGreen-EcR-B1 (Methods). Levels of nuclear-localized mNeonGreen-EcR-B1 correlated with developmental stage, as expected (Fig. S9). Counter to our hypothesis, mNeonGreen-EcR-B1 concentration was largely uniform throughout the fat body (Fig. S10), albeit with some local “patchiness” on the length scale of a few cells. Therefore, despite controlling the average DptA response across larvae over developmental time, these data suggest that EcR-B1 is not responsible for the observed variability in DptA expression within a single larva, though we have not ruled out the role of other EcR components.

**Figure 5:**
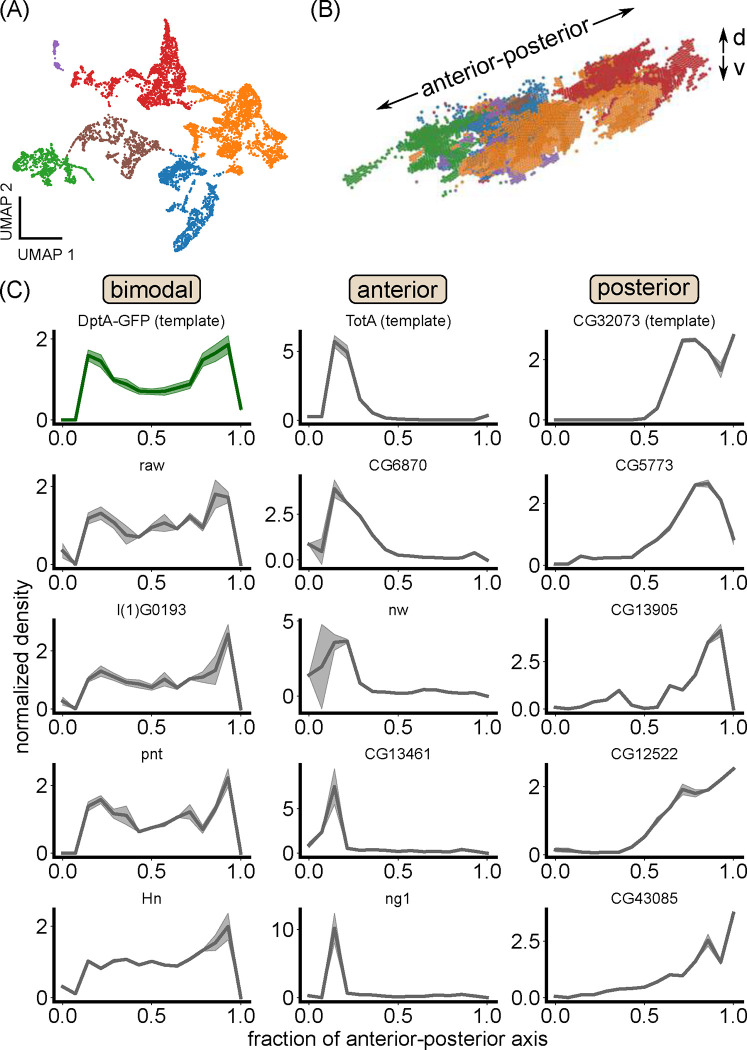
Spatial transcriptomics reveals spatially patterned genes in the larval fat body, including the host-protective factor Turandot-A. (A) UMAP of fat body cells from the early L3 dataset from [30] colored by Leiden clusters. (B) 3D rendering of fat body cells colored by Leiden clusters. Transcriptome clusters correspond to distinct anatomical regions within the fat body. The anterior-posterior and dorsal-ventral (”d-v”) axes are noted. (C) Top genes exhibiting spatial patterning in a bimodal (left), anterior-biased (middle), or posterior-biased fashion. Expression patterns (linear in transcript counts) are normalized so they integrate to one. The top row of genes were used as templates to extract other genes with similar expression patterns via the Wasserstein-1 distance (Methods). For bimodal genes, the mean DptA-GFP fluorescence intensity pattern was used as a template.

To take a more unbiased approach to defining the spatial microenvironments of the fat body, we analyzed previously published, single-cell resolution spatial transcriptomics data of an unperturbed, early L3 larva obtained used StereoSeq [30]. In our quality checks (Methods), we found that the dataset accurately reproduced known spatial patterns of genes with posterior enrichment, including the Hox gene *abd-A* (Fig. S12) [48], indicating that the data accurately captures spatial patterning within the fat body. Sub-clustering fat body cells resulted in clusters that mapped to structurally and developmentally distinct tissue regions (Fig. 5A,B). In particular, the anterior-dorsal lobes of the fat body emerged as a transcriptionally-distinct region (Fig. 5B, green region). A straightforward differential expression analysis between the anterior-dorsal lobes and the rest of the fat body resulted in over 1000 differentially expressed genes encompassing a wide range of biological processes (Fig. S11A–C).

Remarkably, one of the top hits for genes that define the anterior-most region of the fat body was Turandot-A (TotA), a phosphatidylserine (PS) lipid-binding protein that protects host cells from antimicrobial-peptide-induced damage and apoptosis [49] (Fig. 5C, top row, middle). Specifically, the peak of TotA expression coincides with the peak of anterior antimicrobial peptide expression, around 20% of the anterior-posterior axis. To find more genes that matched this and other specific expression patterns, we used a template-based approach and the Wasserstein-1 distance as a measure of distance between spatial distributions [50] (Methods). We found a large panel of genes with bimodal, anterior-biased, and posterior-biased expression patterns (Fig. 5C). Many of the top hits were genes of unknown function. For annotated genes, no clear trend in function emerged. However, one top hit for bimodal genes is l(1)G0193, or orion, which, like TotA, is also a PS lipid-binding protein, one that regulates phagocytic clearance of neurons [51]. Together, these results support the notion of the larval fat body being strongly spatially structured, with transcriptionally-distinct regions along the anterior-posterior axis. Further, we identified the production of PS lipid-binding proteins as a correlate of enhanced antimicrobial peptide production.

## Discussion

Using a live imaging approach, we discovered the existence of spatial microenvironments within the larval fat body that have different levels of antimicrobial peptide production. Regions of high antimicrobial peptide expression correlated with microbial localization via fluid flows in the circulatory system. This observation led us to hypothesize, incorrectly, that the expression pattern was due purely to variations in microbial input, rather than to pre-existing heterogeneity within the fat body. However, loss of blood flow by heartbeat disruption had no effect on the spatial patterning of DptA expression. Therefore, we interpret the data as pointing to a “priming” effect, where the anterior and posterior lobes of the fat body are predisposed to high levels of antimicrobial peptide production.

We speculate that these regions are primed for immune response because they sit in regions of high microbial exposure via blood flow, consistent with the notion of “functional integration” between circulatory and immune systems [33], and analogous to the concentration of leukocytes at the portal vein of the mammalian liver [31]. This spatial configuration also resembles the structure of lymph nodes, where sentinel macrophages line the lymph node interface and upon infection rapidly relay signals to adjacent lymphocytes [1]. In addition to mirroring patterns of blood flow, proximity to key organs (especially for the anterior lobes, which sit near the central nervous system, imaginal disks, and other important structures), may also explain the spatial compartmentalization of immune activity.

Our finding that, within the spatial transcriptomics data from [30], TotA expression is strongly biased to the anterior fat body, where antimicrobial peptide expression is generally the strongest, suggests an intriguing co-regulation mechanism for minimizing self-damage during immune response. This situation conceptually resembles the landscape of the intestine, where sentinel dendritic cells monitor microbial activities in the gut and can induce both proinflammatory and tolerogenic responses [32]. All together, these observations point to the general principle that spatial patterning of immune responses largely reflects the physical structure of the tissue environment, which shapes the statistics of microbial encounters.

Further supporting our results is recent work that identified differences in immune activity between the posterior and middle/anterior larval fat body during parasitic wasp infection [48]. Differential RNA seq analysis between dissected tissue regions revealed an upregulation of Toll, JAK/STAT, and GATA pathway components in the posterior at baseline, which may explain our finding of enhanced antimicrobial peptide expression in this region.

One limitation of our results is that our observations are confined to the early third instar stage. While this developmental stage is short lived compared to the life of the fly (~1 day compared to ~1 month), it is also one that has a strong susceptibility to infection, given the immersion in fermenting substrates and predation from parasitoid wasps that may result in microbial co-infection [52]. The extent to which the spatial patterning of antimicrobial peptide expression occurs in adult flies has only begun to be explored. Recent work using single-nucleus RNA sequencing revealed multiple subtypes of adult fat body cells with distinct immunological characteristics, though their spatial configuration remains to be determined [53]. Expression of antimicrobial peptides following *Providencia rettgeri* infection was largely uniform across cells [53], which suggests that the spatial patterning of antimicrobial peptide expression may be restricted to early larval stages; further testing across microbial stimuli and doses is required for a broader characterization of possible expression patterns in adult flies.

Finally, we emphasize that the live imaging approach introduced here constitutes a significant improvement in the ability to quantify gene expression dynamics during immune responses with a large field of view and single-cell resolution, for any organism. Previous pioneering examples in flies [43] and zebrafish [54] established in vivo, single-cell imaging of fluorescent reporters of gene expression during infection and immune cell differentiation, but were limited to only a few cells at a time. With light sheet fluorescence microscopy, we are able to image over 1000 cells for several hours at 2 minute intervals, significantly expanding the possibility of studying organism-scale immune response dynamics at single-cell resolution. Advances in light sheet microscope design that simplify sample mounting [55, 56] will no doubt improve the feasibility and throughput of such measurements.

## Methods

### Fly stocks

Antimicrobial peptide reporter lines were from [26]. Specifically, we obtained DptA-GFP as a kind gift from Neal Silverman; Drosomycin-GFP from Bloomington (BSDC 55707); Attacin-GFP, Cecropin-A1-GFP, Defensin-GFP, Drosocin-GFP, and Metchnikowin-GFP were kind gifts from David Bilder and Stephan Gerlach. Fat body Gal4 drivers were r4-Gal4 and cg-Gal4, kind gifts from David Bilder. Membranes were marked with UAS-mCD8-mCherry (BDSC 27391). Histones were marked with UAS-His-RFP, a kind gift from Jack Bateman. The larval heart Gal4 driver was NP1029-Gal4, a kind gift from Rolf Bodmer and Erick Eguia. Heartbeat knockdowns were done with UAS-Mhc-RNAi (BDSC 26299) and UAS-Ork1DeltaC (BDSC 8928).

### Generation of mNeonGreen-EcR-B1

The endogenous mNeonGreen-EcR-B1 fusion line was generated by CRISPR-Cas9 genome editing. A DNA mixture was prepared containing 500 ng/μl of mNeonGreen-EcR-B1 homology donor (pTG614), 500 ng/μl of Halo-EcR-B1 homology donor (pTG609; not used in the present study), 300 ng/μl of EcR-B1 U6-sgRNA plasmid (pTG613), and 200 ng/μl of ebony control U6-sgRNA plasmid (pTG625). This mixture was injected by Rainbow Transgenic Flies, Inc. (Camarillo, CA) into a fly line containing a germline-expressed nos-Cas9 transgene at the attP2 locus (chromosome 3). Injectants were crossed to the Sp/CyO; Dr/TM3,ebony(−) double-balancer line, and progeny from vials containing ebony(−)/TM3,ebony(−) flies were crossed to Sp/CyO to establish balanced lines. Successful insertion of mNeonGreen was confirmed by PCR and Sanger sequencing, and the knock-in was made homozygous. The ebony control U6-sgRNA plasmid was a kind gift of Colleen Hannon, and the empty U6-sgRNA plasmid was a kind gift of Mike Stadler.

### Bacteria and Yeast

*E. coli* HS-tdTomato [42] was used for all experiments and was a gift from Travis Wiles and Elena Wall. For every experiment, bacteria were grown fresh overnight shaking at 37°C. *S. cerevisiae* strain SK1 (non-flocculating mutant) with marker HTB1-mCherry-HISMX6 was a gift from Tina Sing. Yeast were streaked on YPD plates and grown overnight at 30°C then picked and grown in YPD liquid culture overnight shaking at 30°C. The full genotype of the yeast strain was MATa, ho::LYS2, lys2, ura3, leu2::hisG, his3::hisG, trp1::hisG, flo8 unmarked, amn1(BY4741 allele)unmarked, HTB1-mCherry-HISMX6, GAL3+.

### Fly husbandry and larva collection

Flies were maintained on a standard diet and were not kept on a strict light-dark cycle. For larva collection, flies were placed in a fresh food vial for 24 hours and then kept for 4 days at 25°C. Larvae were collected via flotation using 20% sucrose solution for no more than 5 minutes. Unless otherwise specified, late L2 larvae were identified by anterior spiracle morphology (containing hybrid L2/L3 spiracles) and placed in a fresh food vial for 6 hours at 25°C. Molt to third instar was confirmed after 6 hours, after which larvae were placed in another fresh food vial. Larvae were then stored according to their age treatment. Most experiments had larvae placed in 18°C for 18 hours (“18h-18C”). In all experiments, larvae were handled gently with a fine paintbrush to avoid potential immune response activation via mechanical stimulation [57].

### Larva anesthesia

For injections and imaging, larvae were subjected to ether anesthesia as described by [58]. In brief, an anesthesia chamber was constructed out of a Coplin staining jar filled with cotton balls and a small glass vial. The cotton was supersaturated with diethyl ether inside of a chemical fume hood. A small cage was made out of a cut top of an Eppendorf tube and fine mesh. Larvae were placed in the cage and the cage was placed in the small glass vial within the anesthesia chamber for a prescribed amount of time. For injections, a batch of around 10 larvae were anesthetized for 2 minutes and 15 seconds. For time-lapse imaging, individual larvae were anesthetized for 45 seconds prior to mounting in glue (see below). For endpoint imaging, larvae were fully immobilized using 3 minutes and 45 seconds of anesthesia exposure. We note that in our experience, the effect of the ether anesthetic on larvae could be quite variable, being sensitive especially to larval humidity and density, and so in some cases was adjusted to obtain the desired effect.

### Microinjection

To prep the injection mix, 1 ml of overnight bacteria or yeast culture was centrifuged for 2 minutes in a small centrifuge at 8000 RPM, washed once, and resuspended in 200 *μ*l of 0.2% sterile saline solution. The injection mix contained a 1:1 mix of this bacterial or yeast solution with a 1 mg/ml solution of Cascade Blue Dextran, which acts as a fluorescent marker of injection success.

Microinjections were performed using a Narigishe IM 300 microinjector under an Olympus SZX10 fluorescent stereo-microscope. Fine-tipped quartz glass needles were pulled on a Sutter P-2000 pipette puller using 0.7 mm ID/1.0 mm OD quartz glass needles with filament (Sutter item num. QF100-70-7.5). Pulled needles were filled with injection mix using a micropipette loader tip and then inserted into a needle holder mounted on a 3-axis micromaniuplator. The needle was gently broken on the edge of a glass slide, producing a 5–10 *μ*m sized tip (Fig. S1). We found that quartz glass was required to obtain a needle that was both fine and rigid enough to easily penetrate the larval body wall. Injection droplet size was calibrated to a 300 *μ*m diameter using a sterilized stage micrometer and was periodically checked throughout an injection session.

Batches of around 10 larvae were anesthetized for 2 minutes and 15 seconds and then mounted on a sterilized glass slide dorsal side up. Prior to injection, larva health status was assessed by looking for a normal heartbeat and minor mouth hook movements (for experiments involving loss of heartbeat, just minor mouth hook movements were used as a marker of health). Except for the injection location control experiments (Fig. S2C–F), injections were done on right side of the body wall between segments 5 and 7, avoiding the fat body itself. Needle penetration was done under a low-intensity brightfield light, but then the light sources was switched to a blue fluorescence channel for the actual injection. The needle was held in place for 10 seconds and the blue dye was observed to confirm a normal flow pattern: the dye as a bulk shifts to the posterior and then dye can be seen being pumped through the larval heart. Animals with abnormal flow patterns were discarded, as were any animals for which significant dye leaked out of the injection site after needle removal. After successful injection, larvae were placed in a humid Petri dish. Using this injection method with fine-tipped quartz needles, we observed no melanization response common to other infected wound models.

### Light sheet fluorescence microscopy

Three-dimensional images were acquired using a Zeiss Z.1 Light Sheet Fluorescence Microscope. Two different configurations were used in this paper: (1) 20x/1.0 NA water dipping detection objective with 10x/0.2 NA illumination objectives and (2) 5x/0.16 NA air objective with 5x/0.1 NA illumination objectives. The detection objective used for each experiment is listed below. For all experiments and for each *z*-plane, images were acquired with both excitation sheets in rapid succession and then later averaged. All experiments used pivot scanning to reduce striping artifacts.

### Single time point imaging with the 20x water objective

The 20x water configuration was used for single-time point images only. Larvae were immobilized with ether and embedded in a 1% agarose gel pulled into a glass capillary. Laser power was 5 % maximum for both 488nm and 561nm channels. Exposure time was 30 ms, light sheet thickness was set to 6.5 *μ*m, and *z*-slices were acquired every 2 *μ*m. To capture the full larva width, a zoom of 0.7 was used and the light sheet thickness was extended to 6.5 *μ*m. However, at this zoom, the light sheet incompletely filled the detection plane in the vertical direction, leading to low-intensity artifacts at the top and and bottom of images. Therefore, images were cropped in vertical direction. In addition, remaining low-intensity artifacts were corrected by normalizing images by a fit to a reference image obtained by average several pictures of uniform fluorescence (for green fluorescence, a solution of pure EGFP and for red fluorescence, a solution of rhodamine) in agarose. We fit an intensity field of

(1) $$I(x,y)=\frac{I_{0}}{1\;+\;{\left(\frac{x-x_{c}}{x_{R}}\right)}^{2}}e^{-\frac{{\left(y-y_{c}\right)}^{2}}{2\sigma_{y}^{2}}}$$

where x is the sheet propagation direction and y is the vertical direction.

Images taken with the 20x water objective: Fig. 1C, Fig. 4H, J, Fig. S9, Fig. S5D, Fig. S10.

### Time lapse imaging with the 5x air objective

As fly larvae cannot receive sufficient oxygen while submersed in water, imaging of these samples for longer than a few minutes on classical light sheet microscopes, which rely on immersion in a refractive medium, poses a technical challenge. Our solution was to use halocarbon oil as an immersive medium. Halocarbon oil is rich in oxygen and larvae can survive for over 24 hours fully submerged in it, albeit in a reduced oxygen environment. We filled the sealed imaging chamber with halocarbon oil 27 and the 5x air objective was placed outside of glass window of the chamber. Halocarbon oil 27 has a refractive index of 1.4. To align the Zeiss light sheet in this non-conventional imaging media, we used the objective adapter designed for n=1.45 clearing media together with light sheet galvo mirror settings designed for water immersion.

To mount larvae for timelapse imaging, larvae were anesthetized with ether for 45 seconds and then glued ventral side down onto 2 mm acrylic rods, which were mounted into the standard Zeiss light sheet sample holder. The glue used was Elmer’s washable clear glue, as was done in a previous protocol for adult fly imaging [59]. The glue was applied in three layers. First, a thin layer was used as base to secure the larva and let dry for 3–5 minutes. Then, a layer was applied to each side of the larva, making contact between the lateral body wall and the acrylic rod, and let try for 3–5 minutes. Finally, a layer was applied on the dorsal side of the larva, bridging the two lateral glue layers and avoiding the posterior spiracles, letting dry for 3–5 minutes. This gluing method constrained larval movement and produced minimal aberration on the low-NA 5x objective.

Laser power was 30% of maximum for both 488nm and 561nm channels. Exposure time was 30 ms, light sheet thickness was set to 8.16 *μ*m, and *z*-slices were acquired every 4 *μ*m.

Images taken with the 5x air objective: Fig. 2C–D, Fig. 3A, Fig. 4B, Fig. S5A–C, Fig. **??**.

### Widefield microscopy

Low magnification, widefield images of antimicrobial peptide expression patterns were obtained on a Zeiss AxioZoom fluorescence microscope. Larvae were immobilized with ether, mounted on a glass slide dorsal side up, and imaged using a 1X objective using a zoom of 29.5X, an exposure time of 10ms, and an LED power of 100% on an XCite light source.

### Image analysis

#### Image registration and *zarr* conversion

Images for each time point, tile, and light sheet illumination were saved as separate .czi files and then assembled using custom Python code. Images from the two sheet illuminations were combined with a simple average. For images taken with the 20x objective, each fused *z*-plane was corrected for sheet intensity (see “Light sheet fluorescence microscopy” section above). Images from different tiles were registered using stage coordinates extracted from the .czi file using the aicsimageio package [60]. The final image was saved as a 5-dimensional OME-Zarr file [61].

#### Single-cell DptA-GFP expression levels from a membrane marker

Single-cell DptA-GFP levels from Fig. 1 were quantified in 2D maximum intensity projections. In our initial experiments, we aimed to segment fat body cells based on a fluorescent membrane marker, r4>mCD8-mCherry. However, we found that mCD8-mCherry was additionally localized to the periphery of lipid droplets within fat body, which complicated membrane segmentation. Therefore, we took a manual approach and used the interactive visualizaton program napari [62] to click on cell centers. Before maximum intensity projection, the membrane signal was enhanced using a UNet model from PlantSeg (“2dunet_bce_dice_dx3x”) [63]. GFP Fluorescence intensity was summed within a circle of radius of 6 pixels (≈ 2*μ*m) around the manually-defined cell center.

#### Spatial patterns of DptA-GFP expression along the anterior-posterior axis

To quantify tissue-scale spatial patterns of DptA-GFP expression in the absence of a fat body cell marker, we used Multi-Otsu thresholding of 2D maximum intensity projections. Specifically, we computed 2 Otsu thresholds of log-transformed intensity images, resulting in three image categories with typically well-spaced log-intensity peaks: dim background, bright background, and strong GFP signal. We then thresholded on the strong GFP signal and summed along the short axis of the larva to obtain a 1D intensity distribution along the anterior-posterior axis (Fig. 1D).

#### Quantification of DptA-GFP expression dynamics

In our timeseries imaging experiments, we used a nuclear marker, r4>HisRFP. Due to rapid motion from larval twitching and internal hemolymph flows, nuclei were tracked manually in 2D maximum intensity projections using napari [62]. GFP Fluorescence intensity was summed within a circle of radius 6 pixels (≈ 5.5*μ*m) microns around the manually-defined cell center.

#### Quantification of nuclear-localized Ecdysone receptor levels

Fat body nuclei (r4>HisRFP) were segmented in 3D using straightforward thresholding after Gaussian blur. Parameters were tuned such that *E. coli*-tdTomato, though visible in the images, were not segmented due to being much smaller and dimmer than fat body nuclei. Segmentation was done in Python using the GPU-powered package cucim followed by CPU-based labeling using scikit-image. Ecdysone receptor levels (mNeonGreen-EcR-B1) were then quantified by subtracting local background fluorescence around each nuclei, obtained by averaging the pixel values in a shell around each nucleus of obtained by dilating the nuclear mask by 2 pixels and subtracting the original mask, then summing the green channel fluorescence intensity within each nucleus.

#### Bacteria segmentation

Bacteria were segmented in two phases, similar to the approach of [64]. First, single-cell and small bacterial clusters were identified by Difference of Gaussians filtering and thresholding. Then, larger bacterial clusters, which here often appear to be inside of nephrocytes and hemocytes, are segmented by Gaussian blurring and thresholding. The two masks are computed on the GPU, then combined and resulting mask is used to compute a label matrix on the CPU. We then compute the summed fluorescence intensity of each object in the label matrix and estimate number of bacteria per object by normalizing by the median intensity and rounding up to the nearest integer. We chose the median intensity as a normalization factor based on visual inspection of the images and corresponding fluorescence intensities of each object. We defined planktonic bacteria as clusters with a size less than 3 cells, which we determined by visual inspection to most accurately capture single-cells.

#### Computer specifications

Image analysis was done on a custom-built workstation with an Intel Core i9 11900K processor, GeForce RTX 3070 8GB GPU, and 128 GB RAM running Ubuntu 20.04.

### Heartbeat knockdown experiments

We used two strategies to eliminate the heartbeat. First, following the work of reference [46], we over-expressed the potassium channel Ork1 using the larval heart-specific driver NP1029. This scheme produced robust elimination of the heartbeat (Supplemental Movie 9, left). However, we found that starting approximately 3 hours after either bacteria or mock injections, the heart began beating again and by 6 hours was steadily beating in the majority of larvae (Supplemental Movie 9, right). The mechanism behind this effect is unknown. As this timescale of regaining a heartbeat interfered with our immune response measurements, we turned to a more severe perturbation. Following reference [47], we knocked down myosin heavy chain in the larval heart via NP1029>Mhc-RNAi. We found that this scheme eliminated the heartbeat in a manner robust to injection (Supplemental Movie 7).

For the characterization of bacterial spatial distribution and fluid flows in heartbeat-less animals (Fig. 4, **??**), animals were reared and 25°C and staging was less precise—we simply picked early third instar larvae out of the food.

For the measurement of Diptericin expression in heartbeat-less animals and matched controls, we used a trans-heterozygote scheme described in Supplemental Figure S7. F2 larvae were screened for or against the presence of a heartbeat under a dissection microscope. Animals lacking any detectable GFP expression after infection were discarded. In this experiment, larvae were staged precisely according to the 18 hours post L3 molt at 18°C protocol described above. To maximize the effect of the RNAi while including this period at 18°C, larvae were raised from egg laying to late L2 at 29°C (see schematic in Supplemental Figure S7B). Heartbeats (and lack thereof) were monitored throughout the experiment: before and after molt to L3, before and after injections, and before mounting for imaging. Larvae that were first identified as having no heartbeat but later exhibit some beating were discarded. Larvae that were identified to have a heartbeat but lacked a heartbeat after ether exposure prior to injections were also discarded.

### Quantifying the similarity of spatial patterns using the Wasserstein-1 distance

The Wasserstein-1 distance is a measure of distance between probability distributions [65]. It and related metrics have become useful tools in the analysis of spatial transcriptomics data [66–68]. Conceptually, the more general Wasserstein-p distance measures the minimal cost of morphing one distribution into another, where distance is measured using the *p*-norm; it is related the theory of optimal transport [65]. For one-dimensional probability distributions, the Wasserstein-1 distance has a convenient analytic expression. Given two probability distributions, f(x) and g(x), with cumulative distributions F(x) and G(x), respectively, the Wasserstein-1 distance is given by [65]

(2) $$W_{1}(f,g)=\int dx\left|F\left(x\right)-G\left(x\right)\right|.$$

To use this metric for quantifying the distance between spatial patterns of gene expression, we normalized the 1D expression pattern along the anterior-posterior axis by the total mass, such that the result integrated to unity. With this normalization, we can interpret the expression pattern as a probability distribution over positions that describes the probability that a transcript of a given gene sampled at random from the dataset fell within a given anterior-posterior axis bin. This approach is agnostic to the units of the quantity in question, so can be used to compare the spatial arrangements of diverse variables, such as fluorescence intensity from microscopy images and counts of transcripts.

### Spatial transcriptomics analysis

StereoSeq data, in the form of a processed anndata file, of an unpertrubed, early L3 larva was obtained from [30]. Analysis was done using the scanpy package [69]. Fat body cell annotations were taken directly from [30]. Reads were further filtered to 5% detection. Leiden clustering was performed on Pearson analytic residuals [70] with parameters: $n_{iterations}=2,\;resolution=0.04$. The resolution was chosen by starting with a low value and increasing until the anterior fat body emerged as a cluster. Marker genes for each cluster were found using the Wilcoxon test on log1p-transformed counts using Bonferonni correction.

In our quality checks of the data, we noted that multiple “house-keeping” genes, including Act5C, betaTub56D, and alphaTub84B, exhibited moderately enhanced expression in a region between 0 and 20% along the anterior-posterior axis S11D, more anterior than the anterior-dorsal lobes where we saw enhanced antimicrobial peptide expression, which sits at around 20% along the axis. This observation may reflect global modulation of transcription in fat body cells in this region, or it may be an artifact. To avoid identifying genes that correlated with this uptick in house-keeping gene expression, we focused on genes whose expression pattern peaked at around 20% along the anterior-posterior axis, such as TotA.

Genes with specific spatial expression patterns were found using a template matching approach. The Wasserstein-1 metric was used as a distance measure between 1D expression distributions. To suppress large variance in transcript counts, 1D expression distributions were computed as

(3) $$I(x)=10^{\left\langle l(x)\right\rangle }-1,$$

where

(4) $$l(x)={log}_{10}(C(x)+1)$$

and C(x) represents normalized transcript counts in the anterior-posterior bin centered at x. The average occurs over all fat body cells within that bin.

To find genes whose expression pattern mirrors the bimodal antimicrobial peptide expression, DptA-GFP was used as a template. To find anterior-biased genes, TotA was used as a template. TotA was chosen because it is one of the top marker genes defining the anterior fat body cluster, and its expression peak coincides with the observed high expression of antimicrobial peptides upon infection, around 20% of the anterior-posterior axis. In the spatial transcriptomics dataset there is a line of cells annotated as fat body located even more anterior than this region, which we found to exhibited enhanced expression of multiple “house-keeping” genes, including Act5C, betaTub56D, and alphaTub84B S11D. and we were interested in genes that were not peaked in this region. For posterior-biased genes, CG32073 was chosen as a template because it was the leading gene that was down-regulated in the anterior fat body cluster.

Criteria for establishing relevant thresholds and significance values for Wasserstein-1 distances were determined as follows. For DptA-GFP-like patterns, we were were interested in patterns that were closer to DptA-GFP than was a uniform expression profile, which we found to have a Wasserstein-1 distance of 0.07. Therefore, we extracted genes whose Wasserstein-1 distance was within one standard deviation of 0.07. Standard deviations were computed across bootstrapped replicas of the 1D expression distribution. For anterior-biased patterns, to be more stringent in our selection than comparing to a uniform pattern, we used the housekeeping gene, Act5C, as a cutoff. We observed non-uniform patterning in multiple housekeeping genes, including Act5C, that is biased to the anterior fat body (Fig. S11D), peaking in the anterior-most region; indeed Act5C and other housekeeping genes emerge as statistically significant markers of the anterior fat body cluster. While this trend may reflect spatial patterning of total transcriptional activity within the fat body, to keep our analysis conservative, we looked for genes whose Wasserstein-1 distance to TotA was within one standard deviation of the TotA-Act5C distance of 0.14. For posterior-biased genes, we thresholded the Wasserstein-1 distance on a the distance between the template gene CG32073 and a uniform distribution, 0.32.

Tables of genes that result from the standard differential expression analysis and the Wasserstein-1 template matching analysis are included in Supplemental Data Files 1–4.

## Supplementary Material

Supplement 1 **Supplemental Movie 1**: 3D rendering of a “partial response” larva expressing DptA-GFP (green) 24 hours after injection with *E. coli*-tdTomato. The DptA-GFP channel has been log-transformed for visual clarity. Also shown in magenta is a fat body membrane marker, r4>mCD8-mCherry. Anterior is to the left. Scale bar is 500 *μ*m. See also Fig. 1C,i.

Supplement 2 **Supplemental Movie 2**: Timeseries of maximum intensity projections showing the initial activation of DptA-GFP (green). Fat body nuclei are marked in magenta via cg>His-RFP. Movie starts 5 hours post injection with *E. coli*-tdTomato. Anterior is to the left. Scale bar is 500 *μ*m. See also Fig. 3A.

Supplement 3 **Supplemental Movie 3**: Timeseries of maximum intensity projections showing the initial activation of DptA-GFP (green). Fat body nuclei are marked in magenta via cg>His-RFP. Movie starts 6 hours post injection with *E. coli*-tdTomato. Anterior is to the left. Scale bar is 500 *μ*m.

Supplement 4 **Supplemental Movie 4**: 3D rendering of mNeonGreen-EcR-B1 levels (cyan) in fat body nuclei (magenta, cg>His-RFP) 18 hours post molt to L3 at 18°C. Anterior is to the left. Scale bar is 500 *μ*m.

Supplement 5 **Supplemental Movie 5**: 3D renderings of *E. coli*-tdTomato (top, magenta) 3 hours post-injection and the corresponding computational segmentation (bottom, colors). Anterior is to the left. Scale bar is 500 *μ*m.

Supplement 6 **Supplemental Movie 6**: Real time movie of *E. coli*-tdTomato transport in blood flow. Bacteria can be seen being pumped directly through the heart from posterior to anterior (right to left) and then returning via retrograde flow outside the heart (left to right). Anterior is to the left. Scale bar is 250 *μ*m.

Supplement 7 **Supplemental Movie 7**: Real time movies of heartbeats visualized by green autofluorescence in a wild-type larva (left) and in a larva in which myosin was knocked down in the heart (NP1029> Mhc-RNAi). Heart-specific myosin knockdown eliminates the heartbeat but still allows larva motility and body contractions. Anterior is to the left. Scale bar is 250 *μ*m.

Supplement 8 **Supplemental Movie 8**: Real time movie of *E. coli*-tdTomato transport in blood flow. Bacteria can be seen being pumped directly through the heart from posterior to anterior (right to left) and then returning via retrograde flow outside the heart (left to right). Anterior is to the left. Scale bar is 250 *μ*m.

Supplement 9 **Supplemental Movie 9**: Real time movies of hearts visualized by green autofluorescence larvae in which heartbeats were disrupted by heart-specific overexpression of the potassium channel, Ork1 (NP1029>Ork1) [46]. Despite successful elimination of the heartbeat via Ork1 overexpression (left), microinjection with either bacteria (not shown) or mock (right) restarts the heart by 6 hours post-injection. Anterior is to the left. Scale bar is 250 *μ*m.

Supplement 10 **Supplemental Data File 1**: CSV file of genes that are differentially expressed in the anterior fat body (Leiden cluster 2).

Supplement 11 **Supplemental Data File 2**: CSV file of genes that align with the spatial pattern of DptA-GFP expression along the anterior-posterior axis (bimodal).

Supplement 12 **Supplemental Data File 3**: CSV file of genes that align with the spatial pattern of TotA expression along the anterior-posterior axis (anterior).

Supplement 13 **Supplemental Data File 4**: CSV file of genes that align with the spatial pattern of CG32073 expression along the anterior-posterior axis (posterior).

Supplement 14
