# Supplementary material for: Spatial microenvironments tune immune response dynamics in the Drosophila larval fat body
Source: bioRxiv. 2025 Sep 15:2024.09.12.612587. Originally published 2024 Sep 16. Preprint. [Version 2] doi: 10.1101/2024.09.12.612587 (PMC11429692; doi:10.1101/2024.09.12.612587)
Supplement: Supplement 13 [file NIHPP2024.09.12.612587v2-supplement-13.pdf]

bioRxiv preprint doi: https://doi.org/10.1101/2024.09.12.612587; this version posted September 15, 2025. The copyright holder for this preprint (which was not certified by peer review) is the author/funder, who has granted bioRxiv a license to display the preprint in perpetuity. It is made available under aCC-BY 4.0 International license.

Supplemental Movies

Supplemental Movie 1: 3D rendering of a "partial response" larva expressing DptA-GFP (green) 24 hours after injection with *E. coli*-tdTomato. The DptA-GFP channel has been log-transformed for visual clarity. Also shown in magenta is a fat body membrane marker, r4>mCD8-mCherry. Anterior is to the left. Scale bar is 500 μm. See also Fig. 1C,i.

Supplemental Movie 2: Timeseries of maximum intensity projections showing the initial activation of DptA-GFP (green). Fat body nuclei are marked in magenta via cg>His-RFP. Movie starts 5 hours post injection with *E. coli*-tdTomato. Anterior is to the left. Scale bar is 500 μm. See also Fig. 3A.

Supplemental Movie 3: Timeseries of maximum intensity projections showing the initial activation of DptA-GFP (green). Fat body nuclei are marked in magenta via cg>His-RFP. Movie starts 6 hours post injection with *E. coli*-tdTomato. Anterior is to the left. Scale bar is 500 μm.

Supplemental Movie 4: Real time movies of heartbeats visualized by green autofluorescence in a wild-type larva (left) and in a larva in which myosin was knocked down in the heart (NP1029>Mhc-RNAi). Heart-specific myosin knockdown eliminates the heartbeat but still allows larva motility and body contractions. Anterior is to the left. Scale bar is 250 μm.

Supplemental Movie 5: Real time movie of *E. coli*-tdTomato transport in blood flow. Bacteria can be seen being pumped directly through the heart from posterior to anterior (right to left) and then returning via retrograde flow outside the heart (left to right). Anterior is to the left. Scale bar is 250 μm.

Supplemental Movie 6: 3D renderings of *E. coli*-tdTomato (top, magenta) 3 hours post-injection and the corresponding computational segmentation (bottom, colors). Anterior is to the left. Scale bar is 500 μm.

Supplemental Movie 7: Real time movie of *E. coli*-tdTomato transport in the hemolymph of a larva lacking a heartbeat. Bacteria can be seen being pushed around by body wall contractions, but are not pumped through the heart. Loss of heartbeat was achieved by knocking down myosin heavy chain (Mhc) in the heart via the genotype NP1029-Gal4; UAS-Mhc-RNAi; +. Anterior is to the left. Scale bar is 250 μm.

Supplemental Movie 8: Real time movies of hearts visualized by green autofluorescence larvae in which heartbeats were disrupted by heart-specific overexpression of the potassium channel, Ork1 (NP1029>Ork1) [45]. Despite successful elimination of the heartbeat via Ork1 overexpression (left), microinjection with either bacteria (not shown) or mock (right) restarts the heart by 6 hours post-injection. Anterior is to the left. Scale bar is 250 μm.

bioRxiv preprint doi: https://doi.org/10.1101/2024.09.12.612587; this version posted September 15, 2025. The copyright holder for this preprint (which was not certified by peer review) is the author/funder, who has granted bioRxiv a license to display the preprint in perpetuity. It is made available under aCC-BY 4.0 International license.

Supplemental Movie 9: 3D rendering of mNeonGreen-EcR-B1 levels (cyan) in fat body nuclei (magenta, cg>His-RFP) 18 hours post molt to L3 at 18°C. Anterior is to the left. Scale bar is 500 μm.

bioRxiv preprint doi: https://doi.org/10.1101/2024.09.12.612587; this version posted September 15, 2025. The copyright holder for this preprint (which was not certified by peer review) is the author/funder, who has granted bioRxiv a license to display the preprint in perpetuity. It is made available under aCC-BY 4.0 International license.

Supplemental Data Files

Supplemental Data File 1: CSV file of genes that are differentially expressed in the anterior fat body (Leiden cluster 2).

Supplemental Data File 2: CSV file of genes that are differentially expressed in the posterior fat body (Leiden cluster 3).

Supplemental Data File 3: ZIP of imaging-based data files.

bioRxiv preprint doi: https://doi.org/10.1101/2024.09.12.612587; this version posted September 15, 2025. The copyright holder for this preprint (which was not certified by peer review) is the author/funder, who has granted bioRxiv a license to display the preprint in perpetuity. It is made available under aCC-BY 4.0 International license.

Supplemental Figures

bioRxiv preprint doi: https://doi.org/10.1101/2024.09.12.612587; this version posted September 15, 2025. The copyright holder for this preprint (which was not certified by peer review) is the author/funder, who has granted bioRxiv a license to display the preprint in perpetuity. It is made available under aCC-BY 4.0 International license.

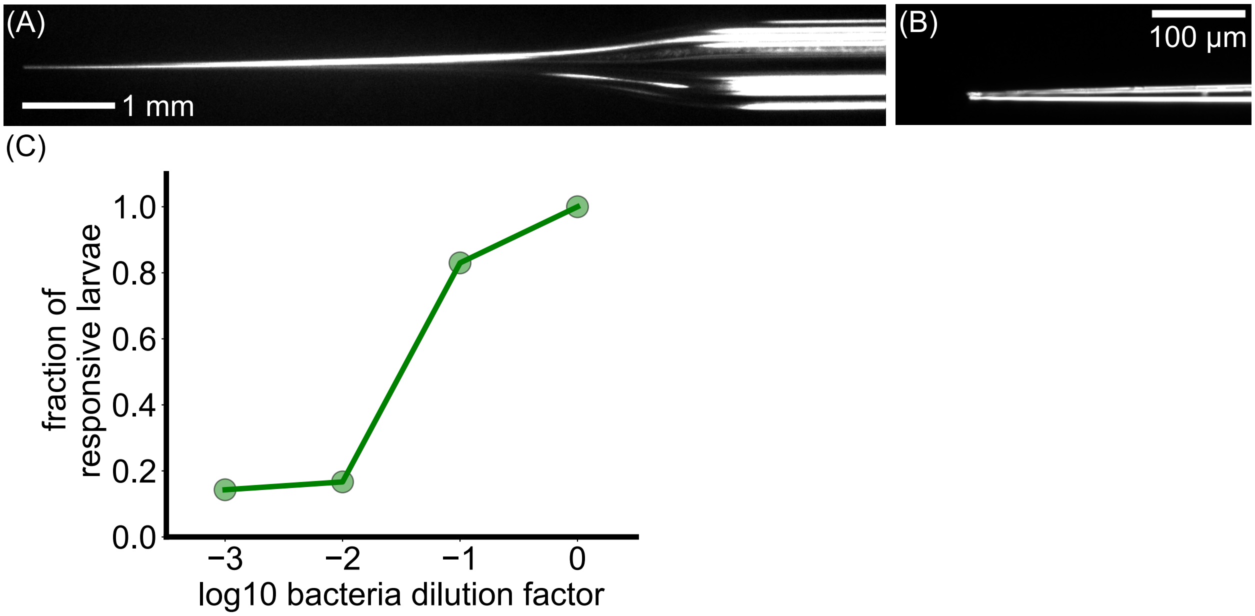

Figure S1: **Details of microinjections.** (A) Brightfield image of an injection needle showing the taper. (B) Brightfield image of the needle tip, which ranges from 5-10 μm. (C) Fraction of larvae showing detectable DptA-GFP expression on a low-magnification widefield microscope as a function of injection dose, in terms of dilution factor of the initial inoculum. The inoculum contains on average 10^5 *E. coli* cells. Number of larvae per group: 7, 18, 12, and 13, for 1000x, 100x, 10x, and 1x dilution factors, respectively.

bioRxiv preprint doi: https://doi.org/10.1101/2024.09.12.612587; this version posted September 15, 2025. The copyright holder for this preprint (which was not certified by peer review) is the author/funder, who has granted bioRxiv a license to display the preprint in perpetuity. It is made available under aCC-BY 4.0 International license.

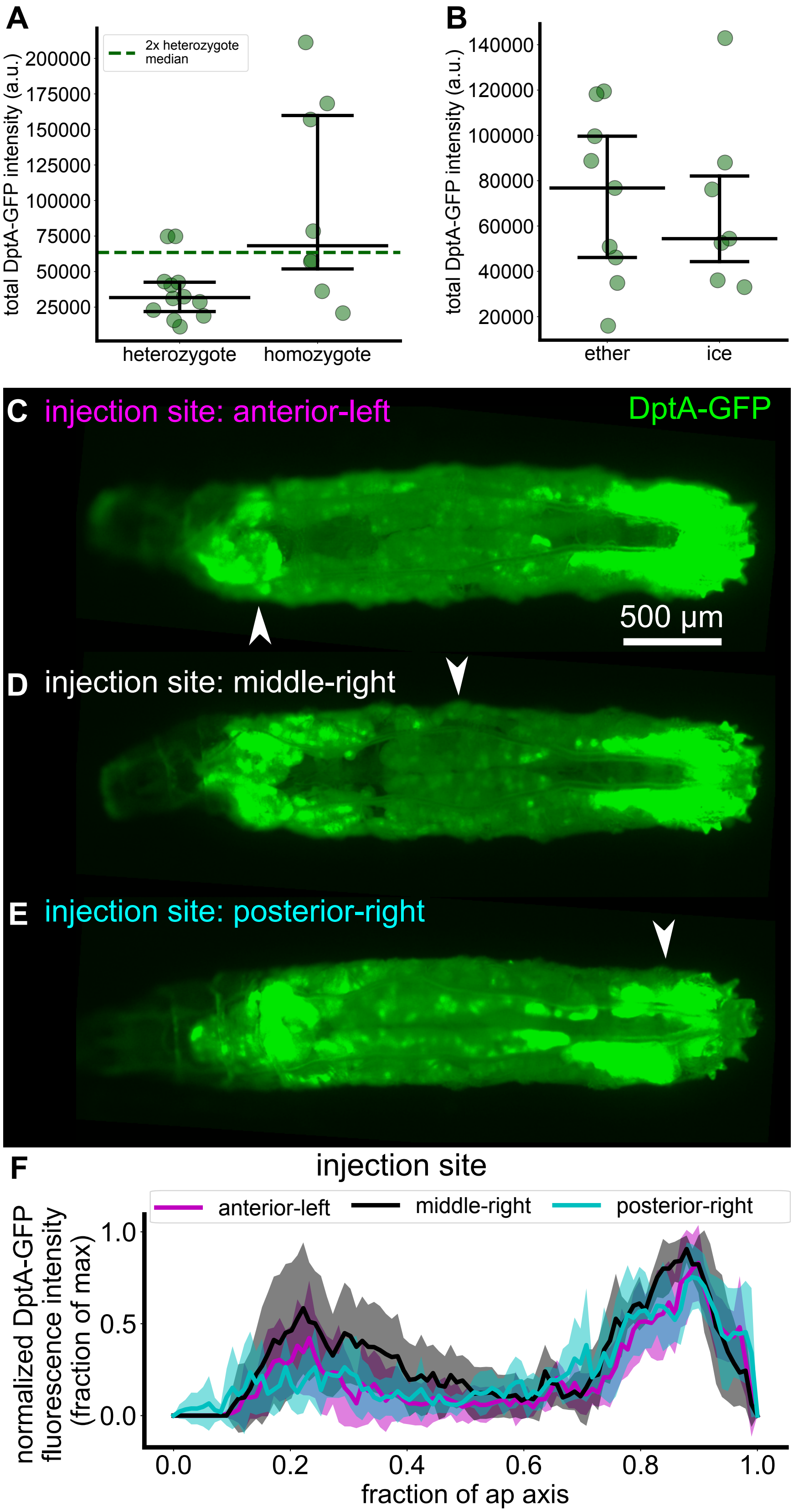

Figure S2: (Caption on next page)

bioRxiv preprint doi: https://doi.org/10.1101/2024.09.12.612587; this version posted September 15, 2025. The copyright holder for this preprint (which was not certified by peer review) is the author/funder, who has granted bioRxiv a license to display the preprint in perpetuity. It is made available under aCC-BY 4.0 International license.

Figure S2: (Previous page.) **Expression of DptA-GFP after microinjection of *E. coli* produces a repeatable, quantitative spatial pattern that is independent of injection site.** (A) Quantification of total DptA-GFP fluorescence intensity 24 hours post infection from a widefield microscope in animal heterozygous and homozygous for the reporter. While there is strong animal-animal variability, the median intensity of homozygotes ($7.4 \cdot 10^4$ a.u.) is close to twice the median intensity of heterozygotes ($3.2 \cdot 10^4$ a.u.), as expected. (B) Total DptA-GFP fluorescence intensity of larvae in which either ether or cold shock ("ice") was used for immobilization during injection. The two immobilization methods produce distributions of total DptA-GFP expression that are comparable within error. (C)-(F) The observed spatial pattern of DptA-GFP expression is independent of injection site. Larvae were injected at 3 different locations, "anterior left", "middle right", and "posterior right" (noted by white arrow heads in the images) and were assessed for DptA-GFP expression 24 hours later on a widefield microscope (single z-plane widefield images shown in (C)-(E), quantification in panel (F), mean and standard deviation of fluorescence intensity normalized to the maximum value for each larva across anterior-posterior bins. N =7 larvae for anterior, 5 for middle, 6 for posterior). The contrasts of images in (C)-(E) were adjusted identically; the apparent saturation does not reflect saturation of the camera, but was chosen to highlight regions of weak expression.

bioRxiv preprint doi: https://doi.org/10.1101/2024.09.12.612587; this version posted September 15, 2025. The copyright holder for this preprint (which was not certified by peer review) is the author/funder, who has granted bioRxiv a license to display the preprint in perpetuity. It is made available under aCC-BY 4.0 International license.

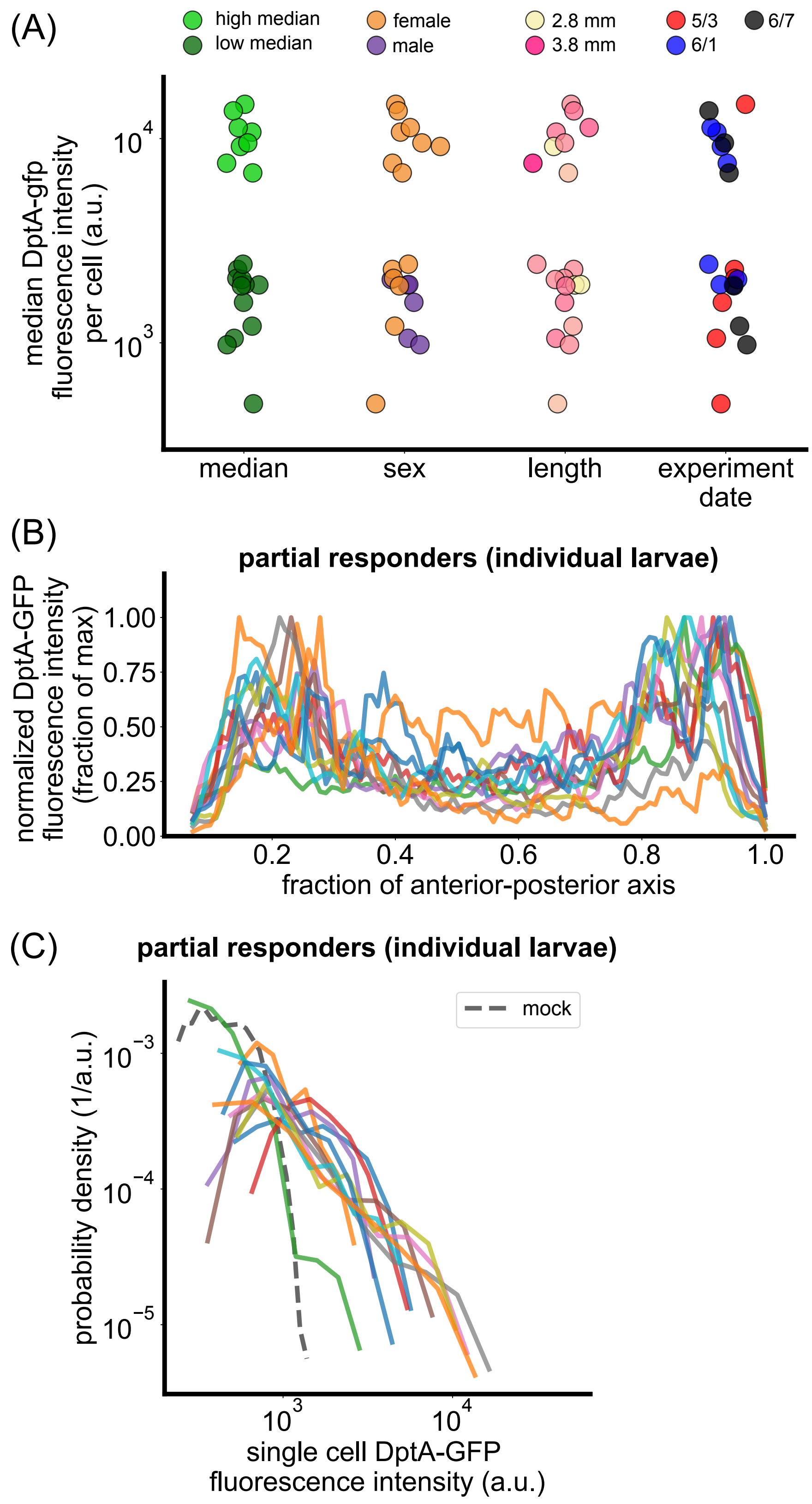

Figure S3: (Caption on next page)

bioRxiv preprint doi: https://doi.org/10.1101/2024.09.12.612587; this version posted September 15, 2025. The copyright holder for this preprint (which was not certified by peer review) is the author/funder, who has granted bioRxiv a license to display the preprint in perpetuity. It is made available under aCC-BY 4.0 International license.

Figure S3: (Previous page.) **Additional details of the partial DptA response.** (A) Partial-complete split by different metadata. From left to right: original clustering by single-cell median DptA-GFP fluorescence intensity produces a clean separation; larva sex, where we see a partial correlation in that all the males observed are partial responders; length of the fat body, which is a proxy for developmental stage and thus Ecdysone levels, though we see no correlation; experiment date, to control for effects related to the details of experiment preparation and injections, where we see no correlation. (B) Normalized DptA-GFP distributions along the anterior posterior axis shown for each larva in the partial responses group. (C) Probability densities of single-cell DptA-GFP fluorescence intensity for each larva in the partial responses group.

bioRxiv preprint doi: https://doi.org/10.1101/2024.09.12.612587; this version posted September 15, 2025. The copyright holder for this preprint (which was not certified by peer review) is the author/funder, who has granted bioRxiv a license to display the preprint in perpetuity. It is made available under aCC-BY 4.0 International license.

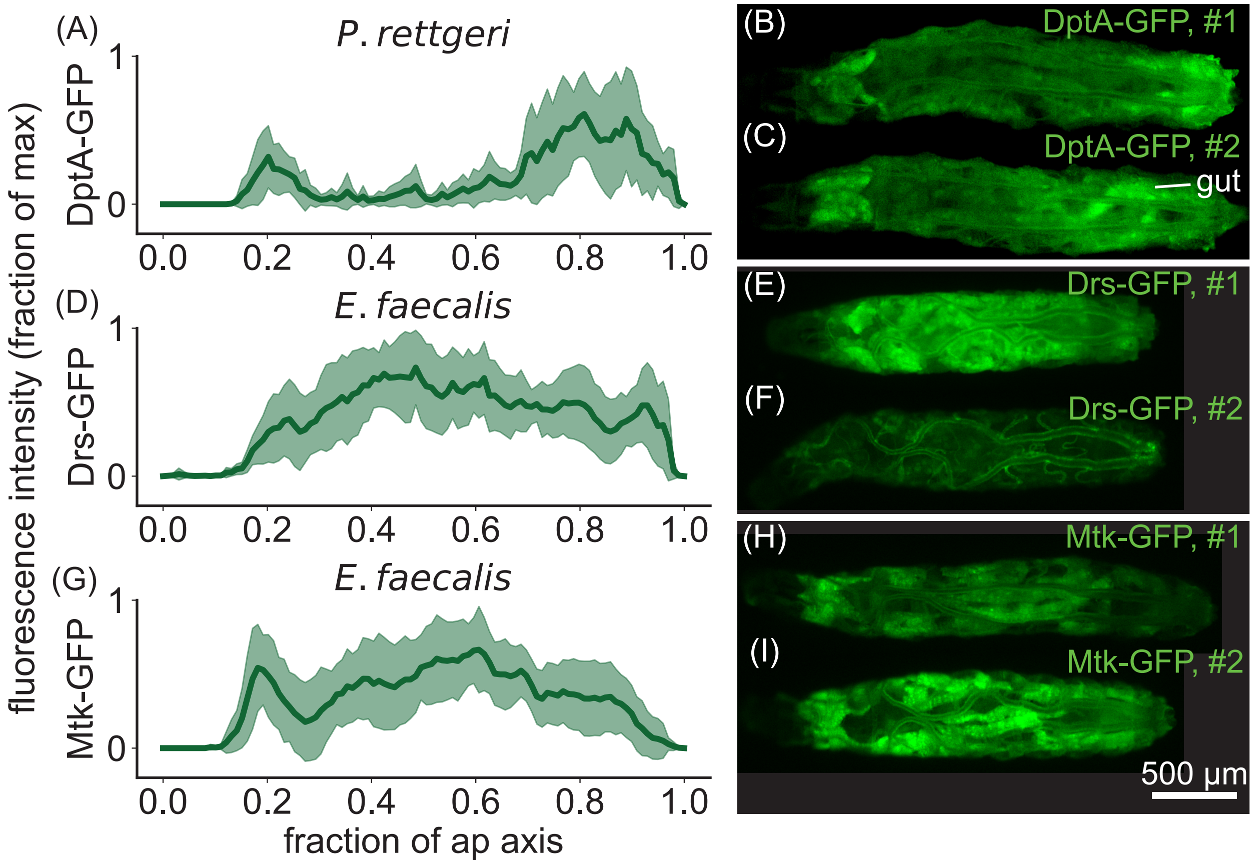

Figure S4: **A spatially heterogeneous AMP response occurs during microinjection-induced infection with *P. rettgeri* but not with *E. faecalis*.** Left panels: fluorescence intensity profiles of AMP reporters along the anterior-posterior axis, normalized to the maximum within each larva and then averaged. Shaded error bars represent standard deviations across larvae. Right panels: widefield microscopy images of two larvae per infection experiment, chosen to represent the diversity of expression patterns observed. (A)-(C) *P. rettgeri* induces a "U-shaped" expression pattern of DptA-GFP on average, mirroring the response to *E. coli* (main text Fig. 1). Some animals exhibit only expression in the anterior fat body (C). (D)-(F) *E. faecalis*, a gram-positive bacteria containing Lys-type peptidoglycan that activates the Toll pathway, induces the Toll-responsive AMP Drosomycin (Drs) in an expression that is homogeneous across the fat body. However, some animals showed weak fat body expression, with the GFP signal being dominated by expression in the tracheal system; all animals were pooled for quantification, which accounts for the slightly different shape of the profile in (D) compared to (G). (G)-(I) *E. faecalis* also induces uniform expression profiles in the Toll-responsive AMP Metchnikowin (Mtk), with less variability across larvae compared to Drs-GFP. All imaging was done 6 hours post injection of bacteria. Approximately 10% of larvae died by this time point within each experiment and were excluded from the analysis.

bioRxiv preprint doi: https://doi.org/10.1101/2024.09.12.612587; this version posted September 15, 2025. The copyright holder for this preprint (which was not certified by peer review) is the author/funder, who has granted bioRxiv a license to display the preprint in perpetuity. It is made available under aCC-BY 4.0 International license.

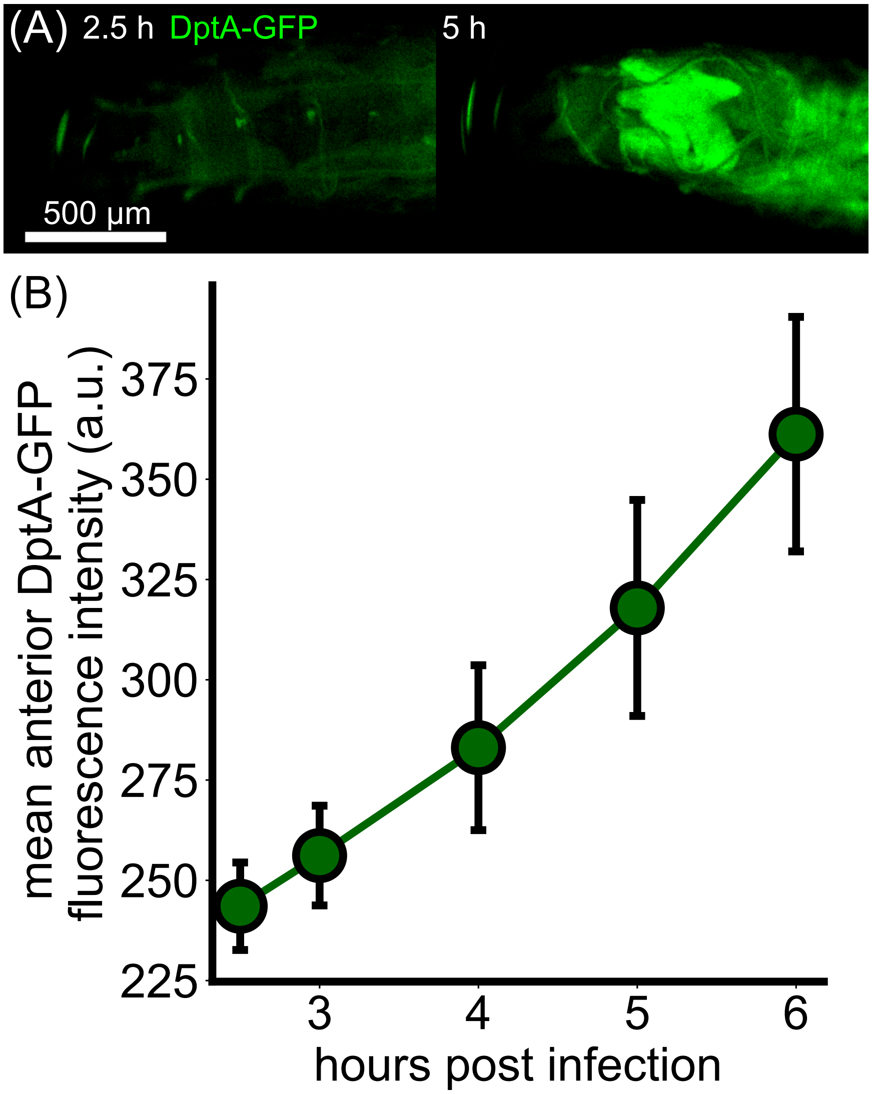

Figure S5: **Dynamics of DptA activation on a conventional widefield microscope mirror findings using light sheet fluorescence microscopy.** (A) Single z-plane images of the anterior of a larva carrying the DptA-GFP reporter taken at 2.5 hours post injection (left) and 5 hours post injection (right). (B) Quantification of DptA-GFP fluorescence intensity in the anterior fat body over time (mean and standard deviation over $N = 11$ larvae).

bioRxiv preprint doi: https://doi.org/10.1101/2024.09.12.612587; this version posted September 15, 2025. The copyright holder for this preprint (which was not certified by peer review) is the author/funder, who has granted bioRxiv a license to display the preprint in perpetuity. It is made available under aCC-BY 4.0 International license.

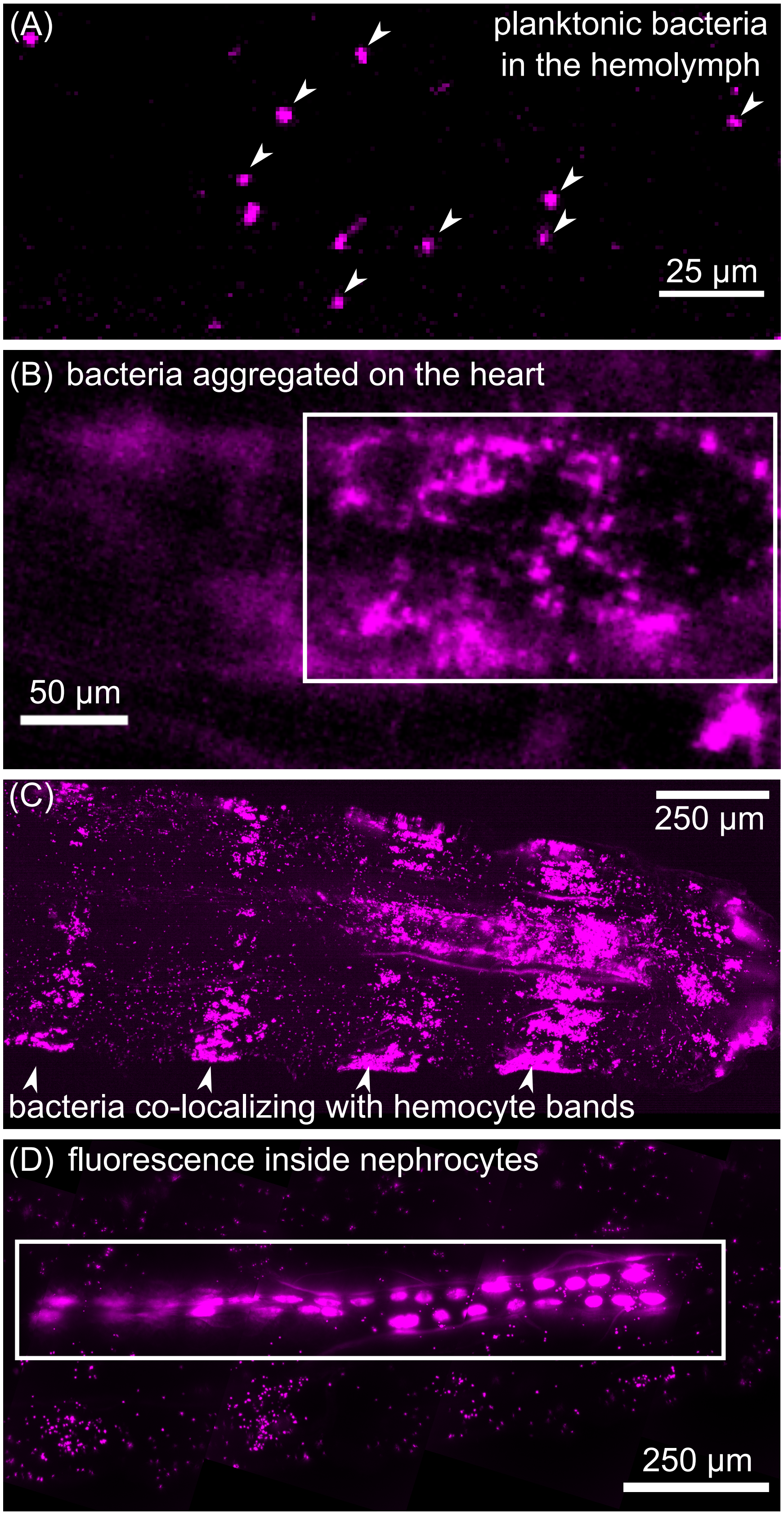

Figure S6: (Caption on next page)

bioRxiv preprint doi: https://doi.org/10.1101/2024.09.12.612587; this version posted September 15, 2025. The copyright holder for this preprint (which was not certified by peer review) is the author/funder, who has granted bioRxiv a license to display the preprint in perpetuity. It is made available under aCC-BY 4.0 International license.

Figure S6: **Gallery of bacterial localization.** (A) Single z-plane image planktonic bacteria (*E. coli*, marked by tdTomato) in the hemolymph (arrow heads). These cells were identified as suspended freely in the hemolymph by their motion in subsequent z-planes. (B) Single z-plane image showing *E. coli* on the posterior end of the heart. (C) Maximum intensity projection image showing an example of *E. coli* co-localizing with known patterns of sessile hemocyte bands (arrow heads) [64]. (D) Maximum intensity projection image showing an example of *E. coli* internalized by nephrocytes embeded in the heart. Images in panels (A)-(C) are from 3-5 hours post injection. Panel (D) is from 6-8 hours post injection.

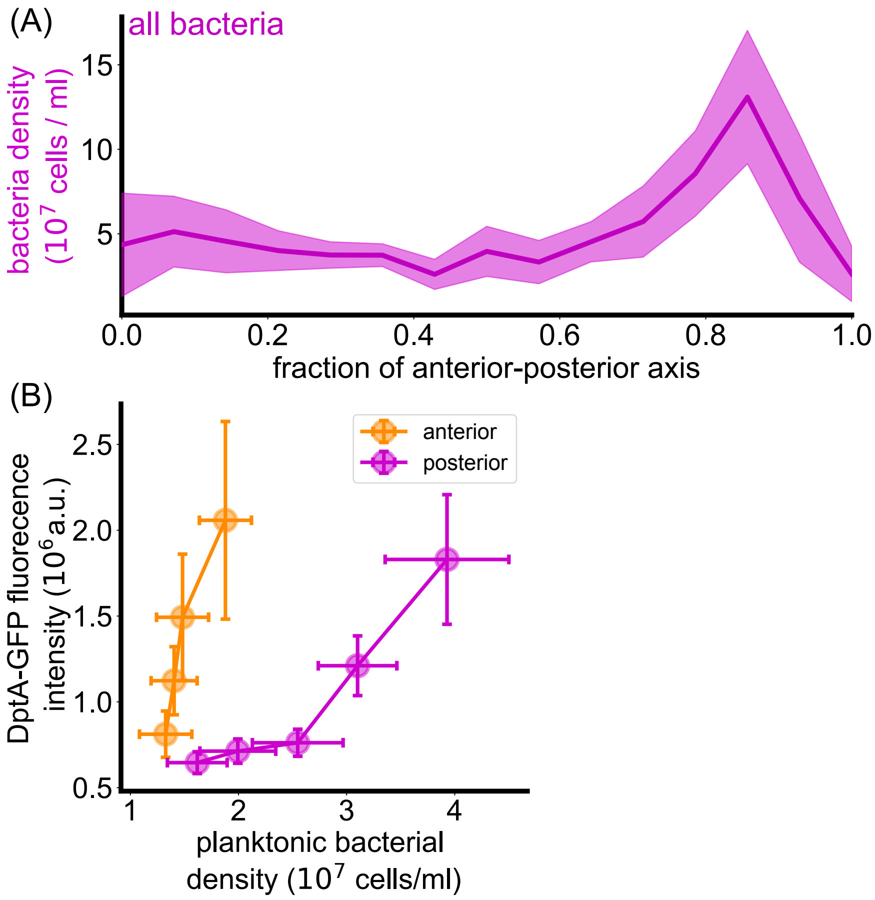

Figure S7: **Additional details of bacteria spatial distribution quantification**. (A) Spatial distribution of all bacteria. (B) Input-output functions for DptA-GFP vs. planktonic bacterial density for the anterior (orange) and posterior (magenta) fat body. The two regions were defined from the peak of DptA-GFP expression to the middle of the fat body, with one anterior-posterior axis bin 0.07x the length of the fat body, or around 160 μm) separating the regions.

bioRxiv preprint doi: https://doi.org/10.1101/2024.09.12.612587; this version posted September 15, 2025. The copyright holder for this preprint (which was not certified by peer review) is the author/funder, who has granted bioRxiv a license to display the preprint in perpetuity. It is made available under aCC-BY 4.0 International license.

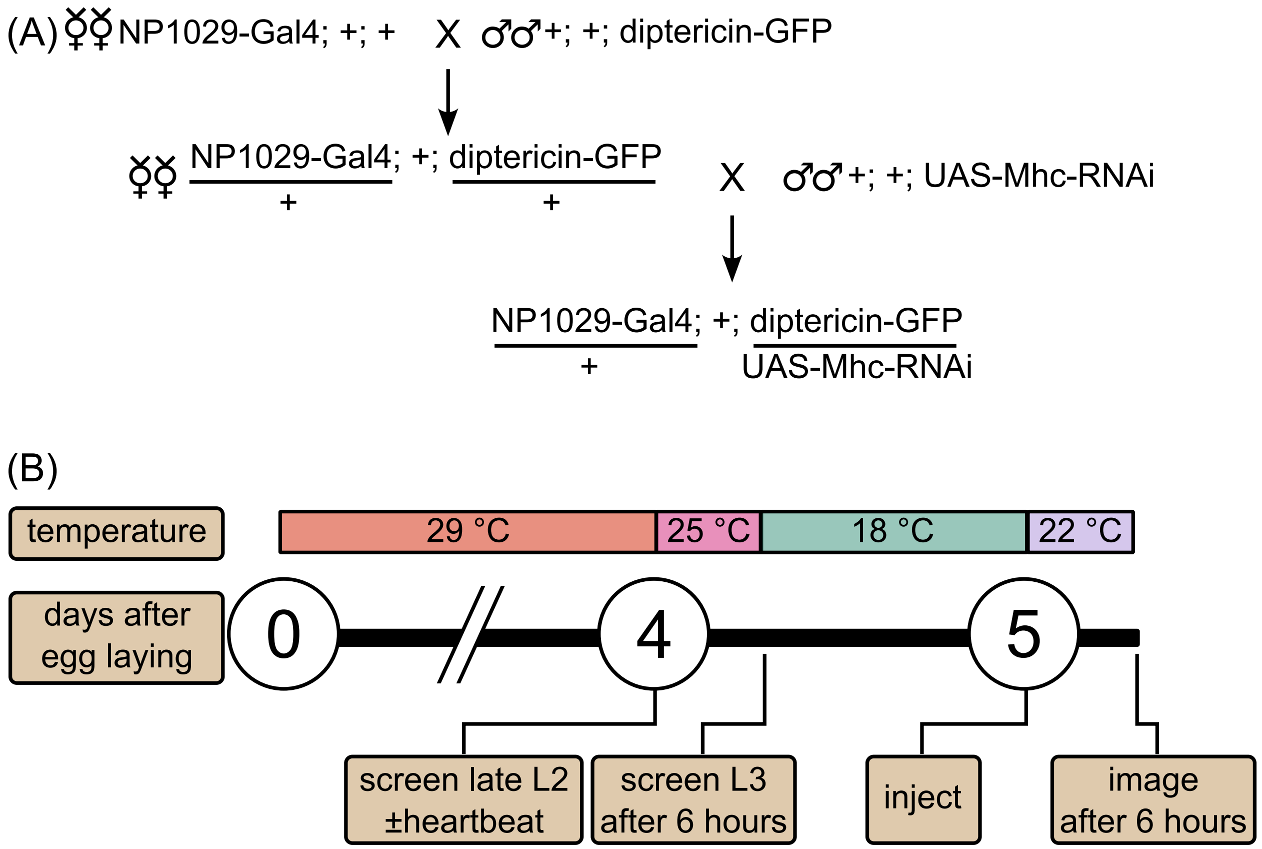

Figure S8: **Details of the heartbeat knockdown experiment.** (A) Fly crossing scheme for generating flies lacking a heartbeat and containing the DptA reporter. (B) Schematic of the timeline and temperatures used in the heartbeat knockdown experiment.

bioRxiv preprint doi: https://doi.org/10.1101/2024.09.12.612587; this version posted September 15, 2025. The copyright holder for this preprint (which was not certified by peer review) is the author/funder, who has granted bioRxiv a license to display the preprint in perpetuity. It is made available under aCC-BY 4.0 International license.

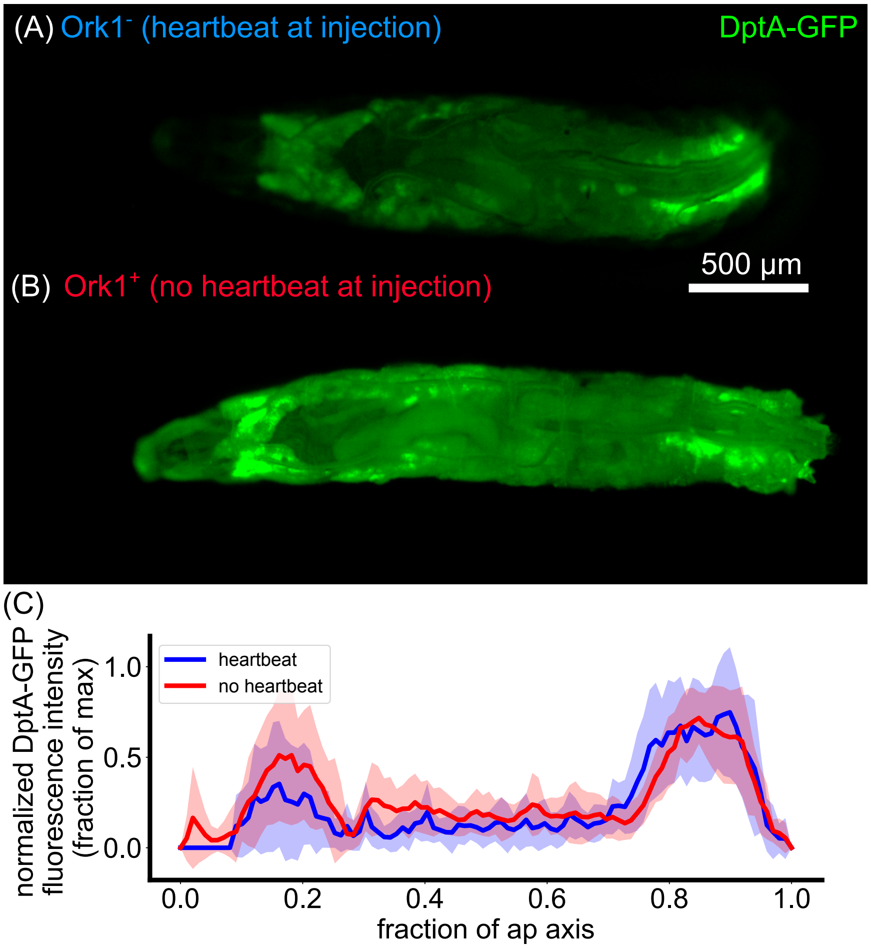

Figure S9: **Temporary loss of heartbeat by overexpression of Ork1 results in no change in the spatial pattern of DptA-GFP expression.** (A)-(B) Single z-plane widefield images of larvae 24 hours after infection for larvae that either had (A) or did not have (B) a heartbeat at the time of injection. Loss of heartbeat was achieved via the larval heart-specific driver NP1029>Ork1 (the crossing scheme was identical to the scheme in Fig. S8A). Starting approximately 3 hours after injection, heartbeats begin to beat again (Supplemental Movie 8). (C) Quantification of DptA-GFP spatial pattern (mean and standard deviation across $N = 6$ larvae for heartbeat$^+$ group, $N = 8$ for heartbeat$^-$ group) normalized to the maximum value for each larva across anterior-posterior bins.

bioRxiv preprint doi: https://doi.org/10.1101/2024.09.12.612587; this version posted September 15, 2025. The copyright holder for this preprint (which was not certified by peer review) is the author/funder, who has granted bioRxiv a license to display the preprint in perpetuity. It is made available under aCC-BY 4.0 International license.

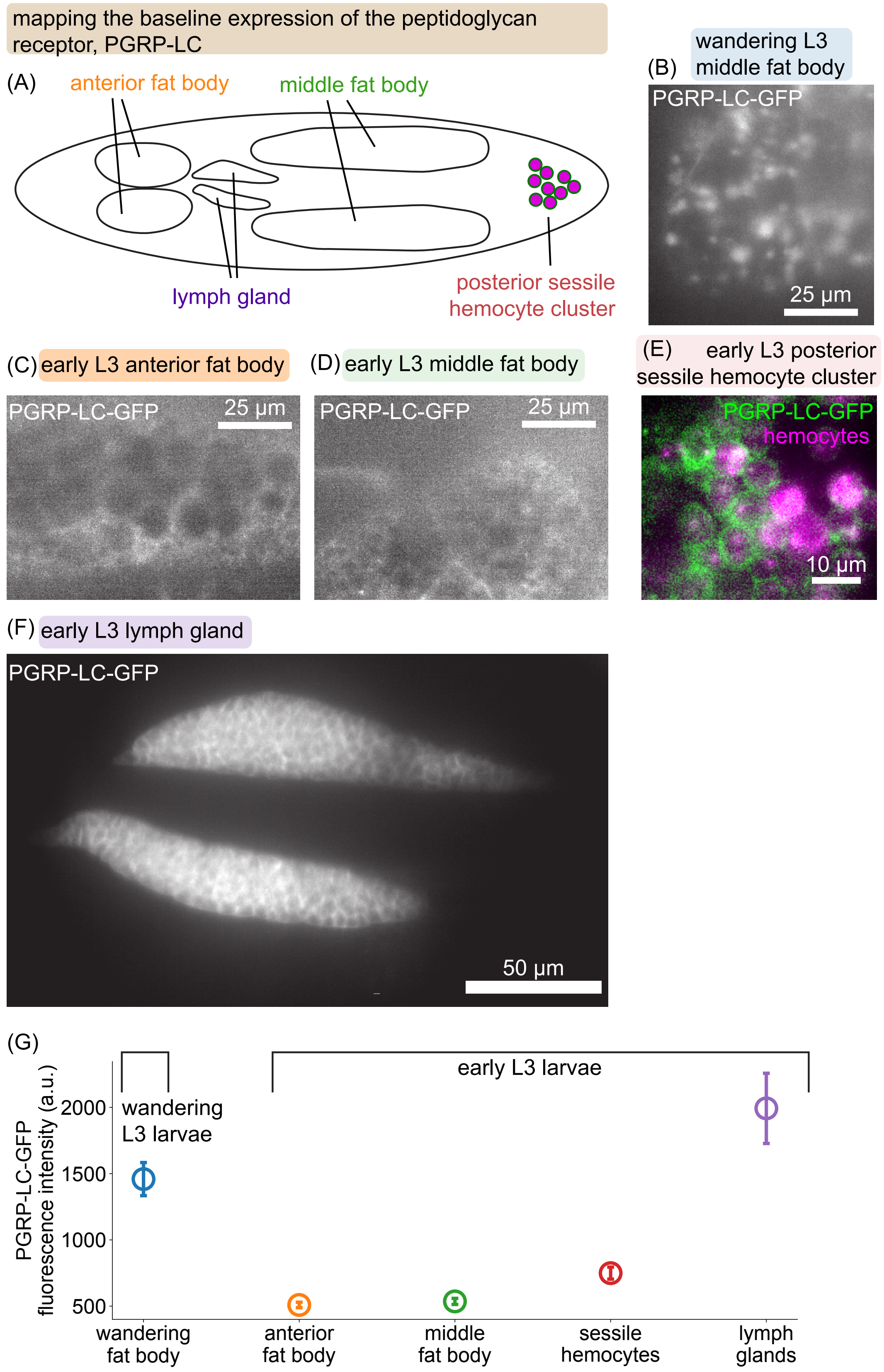

Figure S10: (Caption on next page)

bioRxiv preprint doi: https://doi.org/10.1101/2024.09.12.612587; this version posted September 15, 2025. The copyright holder for this preprint (which was not certified by peer review) is the author/funder, who has granted bioRxiv a license to display the preprint in perpetuity. It is made available under aCC-BY 4.0 International license.

Figure S10: (Previous page.) **Mapping the expression of PGRP-LC-GFP suggests weak but uniform expression of the peptidoglycan receptor across the fat body.** (A) Schematic of immune-relevant tissues in a fly larva. (B)-(F) Maximum intensity projections of PGRP-LC-GFP images taken using light sheet fluorescence microscopy. The fluorescently tagged receptor is the BAC line from [49]. Since expression levels vary widely across tissues, image contrast is adjusted separately for each image for visual clarity (except (C) and (D), which are identical). Quantification of signal intensity is given in (G). (B) Middle fat body of a wandering L3 larva, which shows high levels of expression and protein clusters. (C) Anterior fat body of an early L3 larva (18 hours post molt to third instar at 18°), showing weak expression and fewer detectable clusters. (D) Middle fat body of the same larva in (C), showing similar expression levels as the anterior fat body. (E) We also observe weak PGRP-LC-GFP expression in hemocytes (marked by srpHemo-3xmCherry), which are especially prominent in the posterior sessile clusters [64]. (F) We observe strong expression of PGRP-LC-GFP in the lymph glands. (G) Quantification of fluorescent signal intensity in each of the images in (B)-(F). Signal was extracted via multi-Otsu thresholding with 3 categories—representing image background, tissue background, and tissue signal—and summing pixel intensity in the high intensity category.

bioRxiv preprint doi: https://doi.org/10.1101/2024.09.12.612587; this version posted September 15, 2025. The copyright holder for this preprint (which was not certified by peer review) is the author/funder, who has granted bioRxiv a license to display the preprint in perpetuity. It is made available under aCC-BY 4.0 International license.

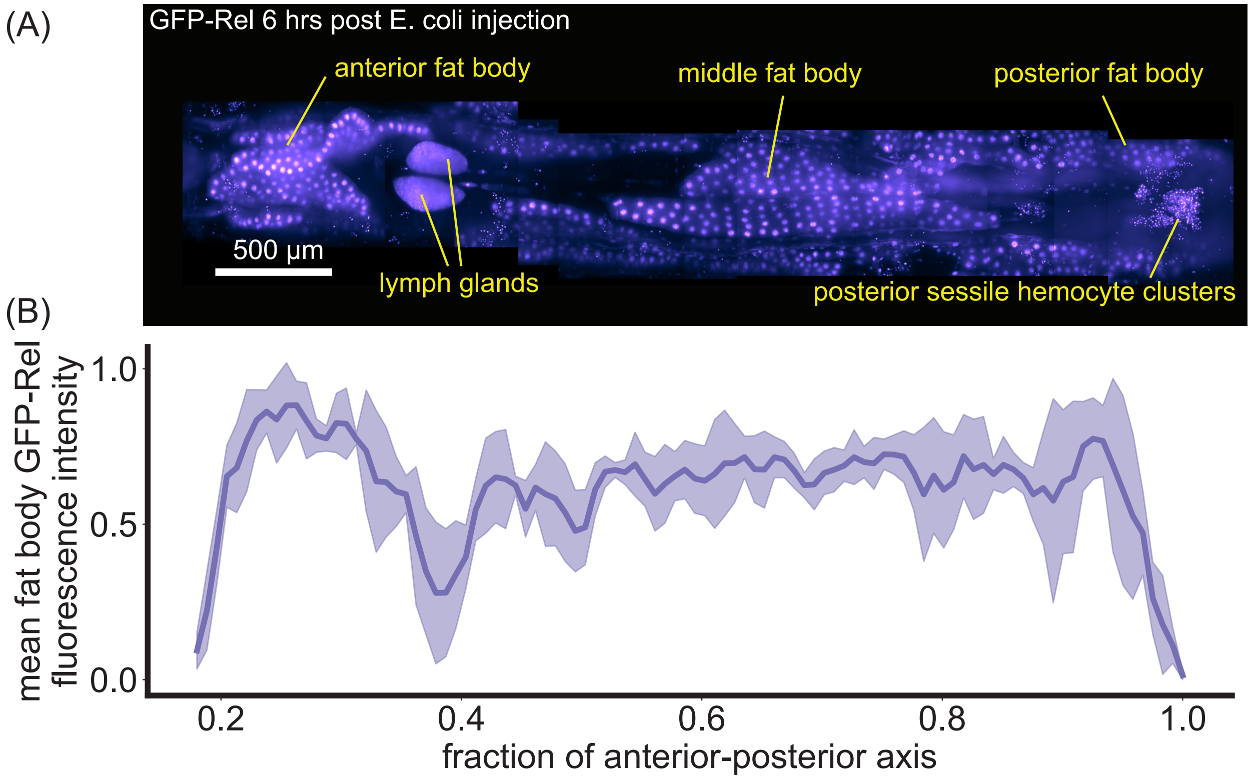

Figure S11: **A transgenic GFP-Relish line shows uniform nuclear localization patterns across the fat body.** (A) GFP-Relish shown as maximum intensity projection from light sheet fluorescence microscopy images 6 hours post infection with *E. coli*. The GFP-Rel construct is in a BAC from [52]. In addition to the fat body, we see activation of GFP-Rel in hemocytes of the lymph gland and posterior sessile clusters. (B) Quantification of GFP-Rel fluorescence intensity (fraction of maximum) along the anterior-posterior axis. Shaded lines indicate standard deviation across $N = 4$ larvae. We used Difference of Gaussians filtering and thresholding to segment fat body nuclei apart from the hemocytes. The profile is uniform along the length of the fat body.

bioRxiv preprint doi: https://doi.org/10.1101/2024.09.12.612587; this version posted September 15, 2025. The copyright holder for this preprint (which was not certified by peer review) is the author/funder, who has granted bioRxiv a license to display the preprint in perpetuity. It is made available under aCC-BY 4.0 International license.

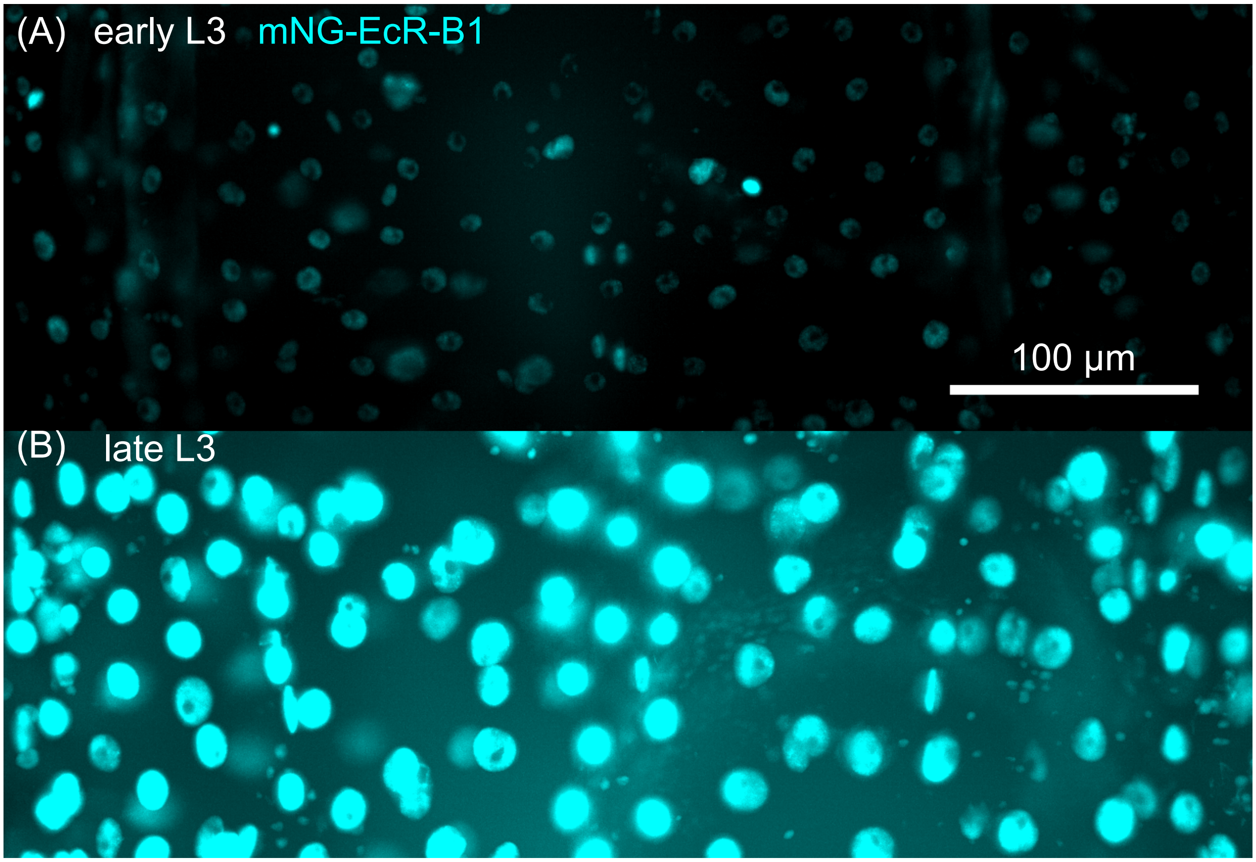

Figure S12: **Nuclear mNeonGreen-EcR-B1 fluorescence intensity correlates with developmental stage** (A)-(B) Maximum intensity projections of mNeonGreen-EcR-B1 images in early (A) and late (B) third instar larvae. Image regions correspond to areas approximately above the anterior-dorsal lobes of the fat body, but the images are not masked by fat body nuclei, so they contain signal from multiple cell types.

bioRxiv preprint doi: https://doi.org/10.1101/2024.09.12.612587; this version posted September 15, 2025. The copyright holder for this preprint (which was not certified by peer review) is the author/funder, who has granted bioRxiv a license to display the preprint in perpetuity. It is made available under aCC-BY 4.0 International license.

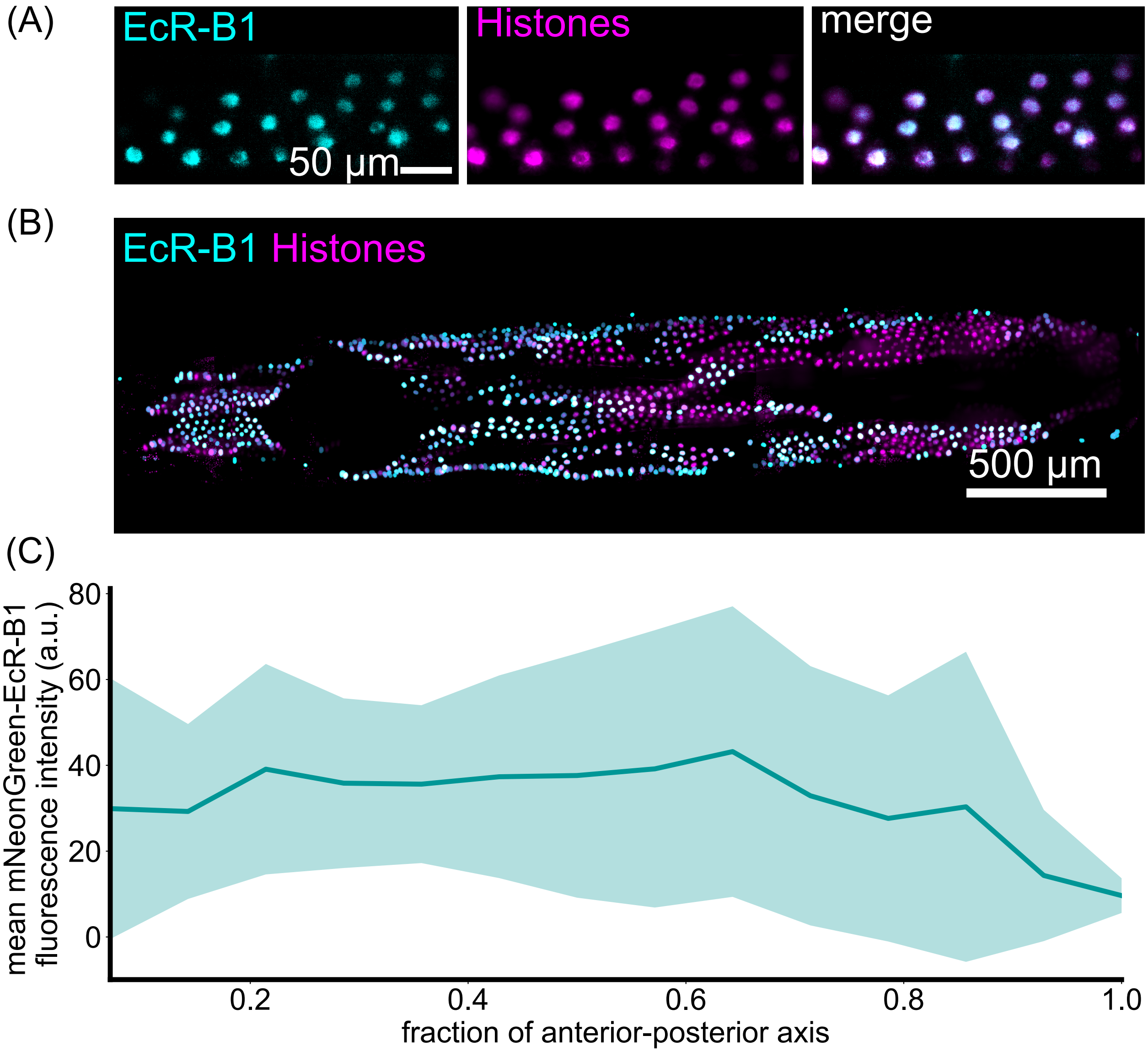

Figure S13: **Nuclear localization of Ecdysone receptor (EcR), a transcriptional regulator of Diptericin, does not correlate spatially with DptA-GFP expression.** (A) An example single z-slice of middle-dorsal fat body nuclei showing raw fluorescence of mNG-EcR-B1 (left, cyan), fat body histones marked by cg-Gal4; UAS-HisRFP (middle, magenta), and the merged image. (B) Maximum intensity projection of a full view of the larval fat body showing mNG-EcR-B1 localization. While the localization pattern exhibits some degree of local structure, unlike DptA-GFP, it is broadly uniform along the anterior-posterior axis. Fat body nuclei were computationally segmented and then false colored (cyan channel) by their mean background-subtracted mNG-EcR-B1 fluorescence intensity (*Methods: Quantification of nuclear-localized Ecdysone receptor levels*). In this way, EcR levels in non-fat body cells are not visualized. Note that cg-Gal4 also labels hemocytes, but hemocytes are computationally removed based on their smaller size (*Methods: Quantification of nuclear-localized Ecdysone receptor levels*). (C) Quantification of nuclear-localized mNG-EcR-B1 levels along the anterior-posterior axis. Solid line and shaded error bars are the mean and standard deviation respectively across $N = 6$ larvae.

bioRxiv preprint doi: https://doi.org/10.1101/2024.09.12.612587; this version posted September 15, 2025. The copyright holder for this preprint (which was not certified by peer review) is the author/funder, who has granted bioRxiv a license to display the preprint in perpetuity. It is made available under aCC-BY 4.0 International license.

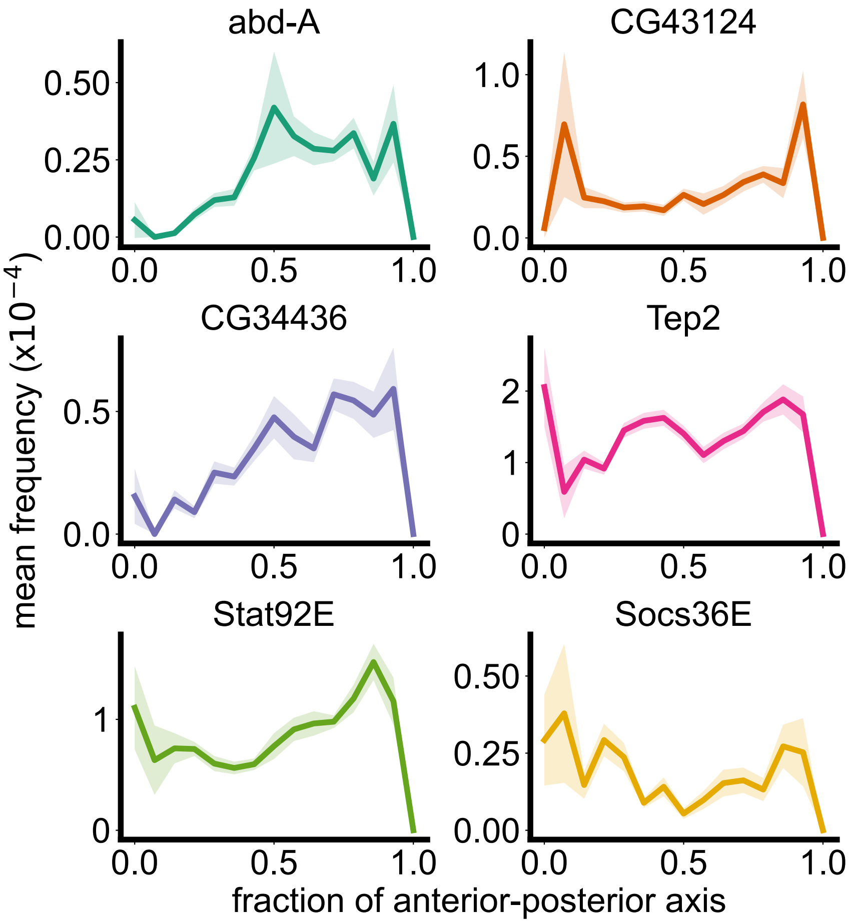

Figure S14: **Spatial transcriptomics data recapitulates known genes with posterior peaks**. These genes were identified in a differential expression analysis of bulk RNA-seq from dissected tissue fragments and being enriched in the posterior compared to the middle-lateral fat body [31]. The first gene, abd-A, is a Hox gene involved in anterior-posterior patterning. Some genes also exhibit a peak in the anterior fat body, which was not included in [31].

bioRxiv preprint doi: https://doi.org/10.1101/2024.09.12.612587; this version posted September 15, 2025. The copyright holder for this preprint (which was not certified by peer review) is the author/funder, who has granted bioRxiv a license to display the preprint in perpetuity. It is made available under aCC-BY 4.0 International license.

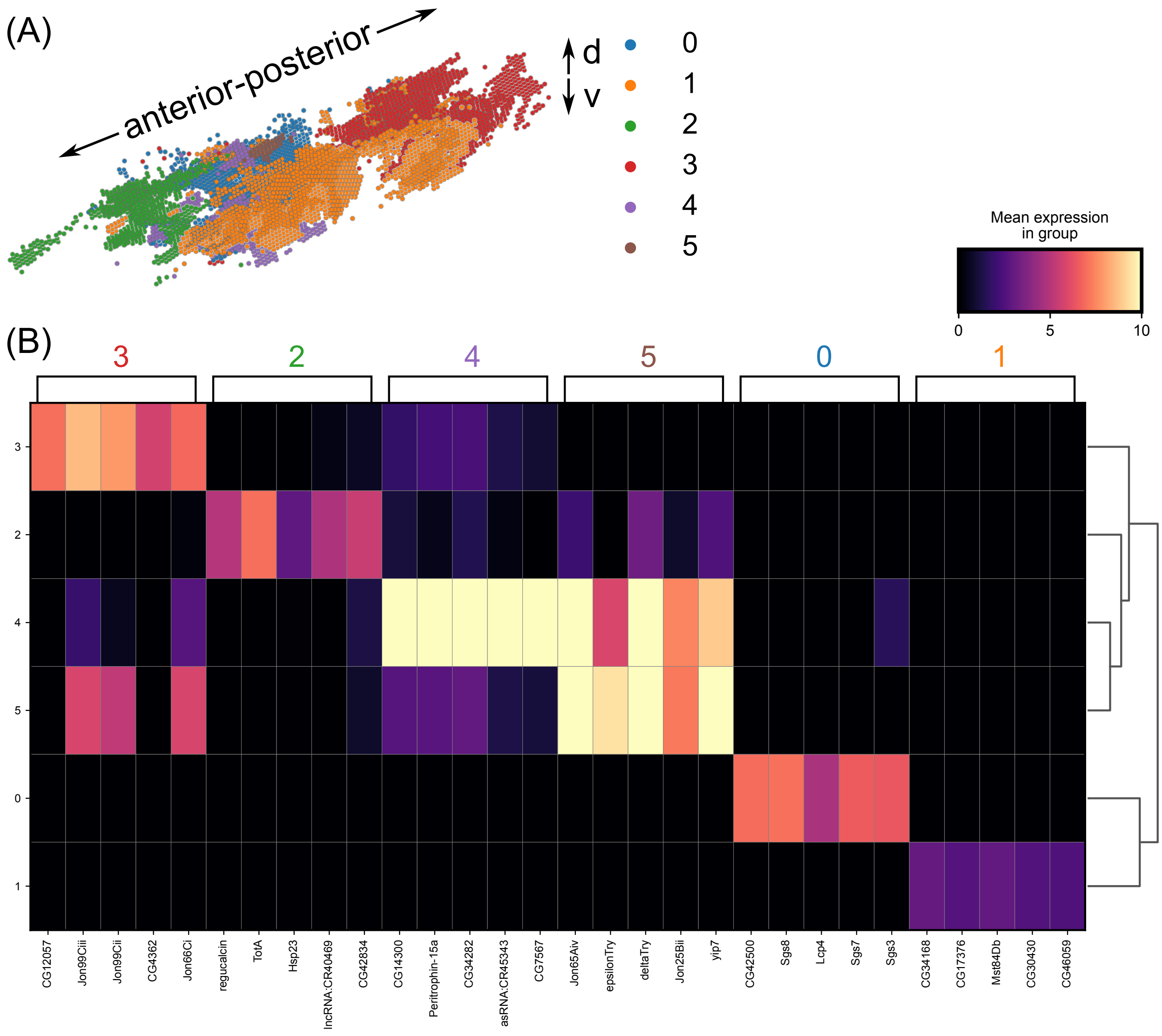

Figure S15: **Additional details of spatial transcriptomics analysis.** (A) 3D rendering of fat body cells colored and labelled by Leiden cluster. (B) Matrix plot showing the top 5 marker genes defining each cluster.
