# Supplementary material for: Spatial microenvironments tune immune response dynamics in the Drosophila larval fat body
Source: bioRxiv. 2024 Sep 16:2024.09.12.612587. Preprint. [Version 1] doi: 10.1101/2024.09.12.612587 (PMC11429692; doi:10.1101/2024.09.12.612587)
Supplement: Supplementary file 1 [file NIHPP2024.09.12.612587v1-supplement-14.pdf]

bioRxiv preprint doi: https://doi.org/10.1101/2024.09.12.612587; this version posted September 16, 2024. The copyright holder for this preprint (which was not certified by peer review) is the author/funder, who has granted bioRxiv a license to display the preprint in perpetuity. It is made available under aCC-BY 4.0 International license.

Supplemental Figures

bioRxiv preprint doi: https://doi.org/10.1101/2024.09.12.612587; this version posted September 16, 2024. The copyright holder for this preprint (which was not certified by peer review) is the author/funder, who has granted bioRxiv a license to display the preprint in perpetuity. It is made available under aCC-BY 4.0 International license.

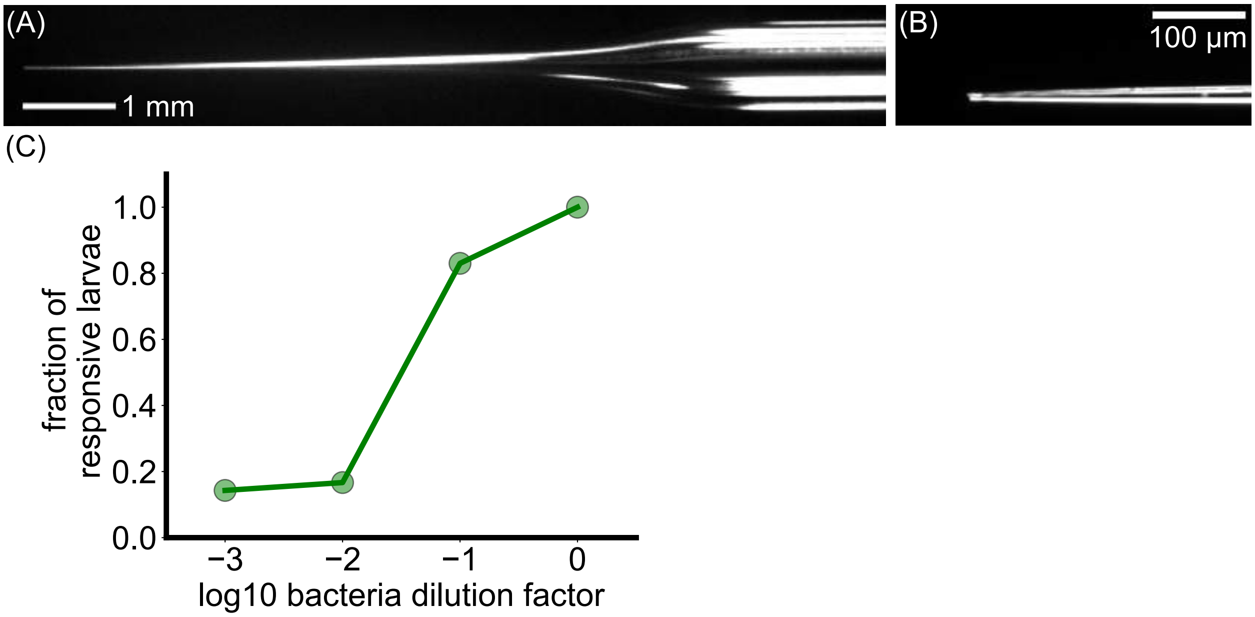

Figure S1: **Details of microinjections** (A) Brightfield image of an injection needle showing the taper. (B) Brightfield image of the needle tip, which ranges from 5-10 μm. (C) Fraction of larva showing detectable DptA-GFP expression on a low-magnification widefield microscope as a function of injection dose, in terms of dilution factor of the initial inoculum. The inoculum contains on average 10^5 *E. coli* cells.

bioRxiv preprint doi: https://doi.org/10.1101/2024.09.12.612587; this version posted September 16, 2024. The copyright holder for this preprint (which was not certified by peer review) is the author/funder, who has granted bioRxiv a license to display the preprint in perpetuity. It is made available under aCC-BY 4.0 International license.

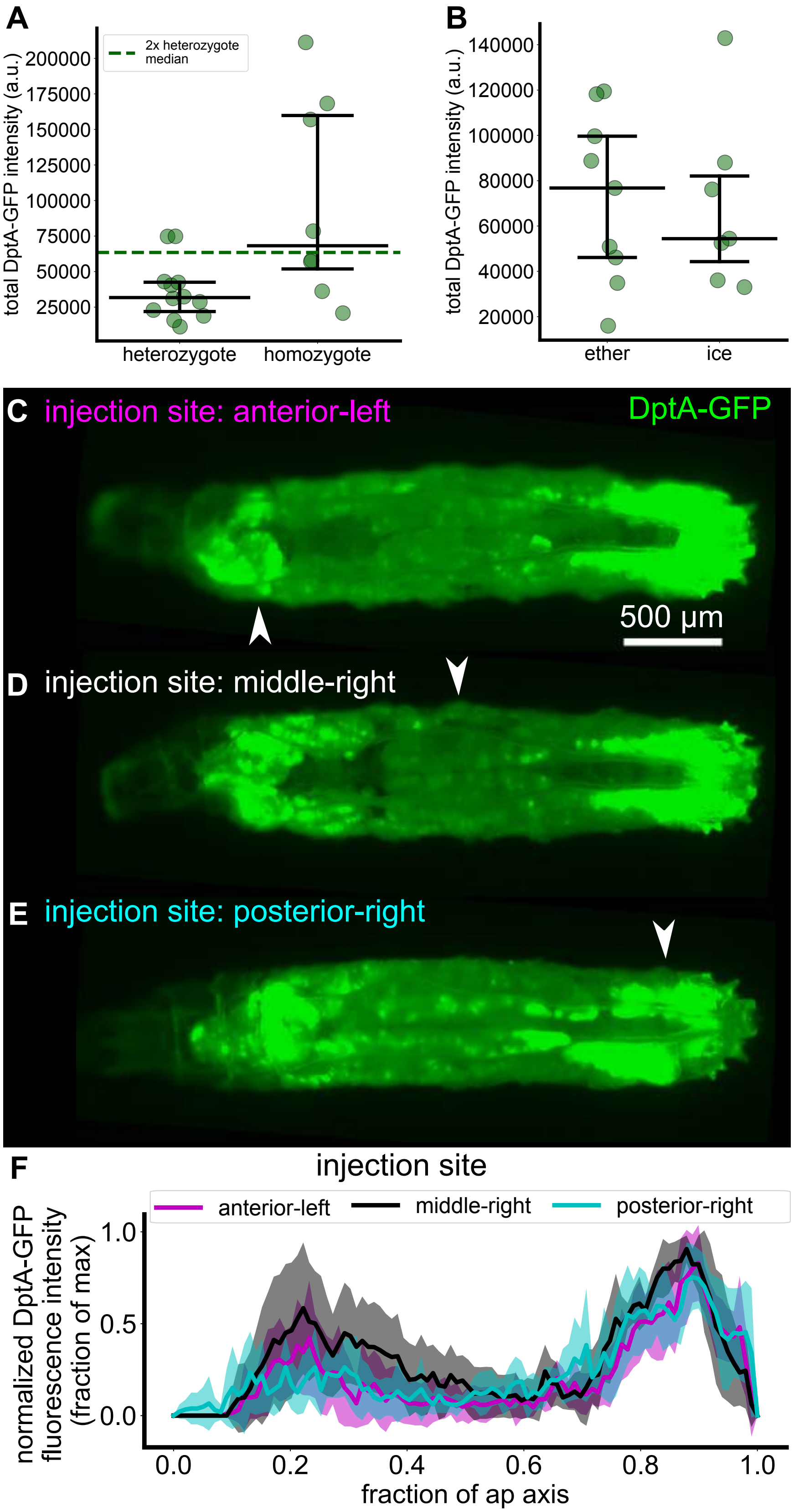

Figure S2: (Caption on next page)

bioRxiv preprint doi: https://doi.org/10.1101/2024.09.12.612587; this version posted September 16, 2024. The copyright holder for this preprint (which was not certified by peer review) is the author/funder, who has granted bioRxiv a license to display the preprint in perpetuity. It is made available under aCC-BY 4.0 International license.

Figure S2: (Previous page.) **Expression of DptA-GFP after microinjection of *E. coli* produces a repeatable, quantitative spatial pattern that is independent of injection site.** (A) Quantification of total DptA-GFP fluorescence intensity 24 hours post infection from a widefield microscope in animal heterozygous and homozygous for the reporter. While there is strong animal-animal variability, the median intensity of homozygotes ($7.4 \cdot 10^4$ a.u.) is close to twice the median intensity of heterozygotes ($3.2 \cdot 10^4$ a.u.), as expected. (B) Total DptA-GFP fluorescence intensity of larvae in which either ether or cold shock ("ice") was used for immobilization during injection. The two immobilization methods produce distributions of total DptA-GFP expression that are comparable within error. (C)-(F) The observed spatial pattern of DptA-GFP expression is independent of injection site. Larvae were injected at 3 different locations, "anterior left", "middle right", and "posterior right" (noted by white arrow heads in the images) and were assessed for DptA-GFP expression 24 hours later on a widefield microscope (single z-plane widefield images shown in (C)-(E), quantification in panel (F), mean and standard deviation of fluorescence intensity normalized to the maximum value for each larva across anterior-posterior bins. $N = 7$ larvae for anterior, 5 for middle, 6 for posterior).

bioRxiv preprint doi: https://doi.org/10.1101/2024.09.12.612587; this version posted September 16, 2024. The copyright holder for this preprint (which was not certified by peer review) is the author/funder, who has granted bioRxiv a license to display the preprint in perpetuity. It is made available under aCC-BY 4.0 International license.

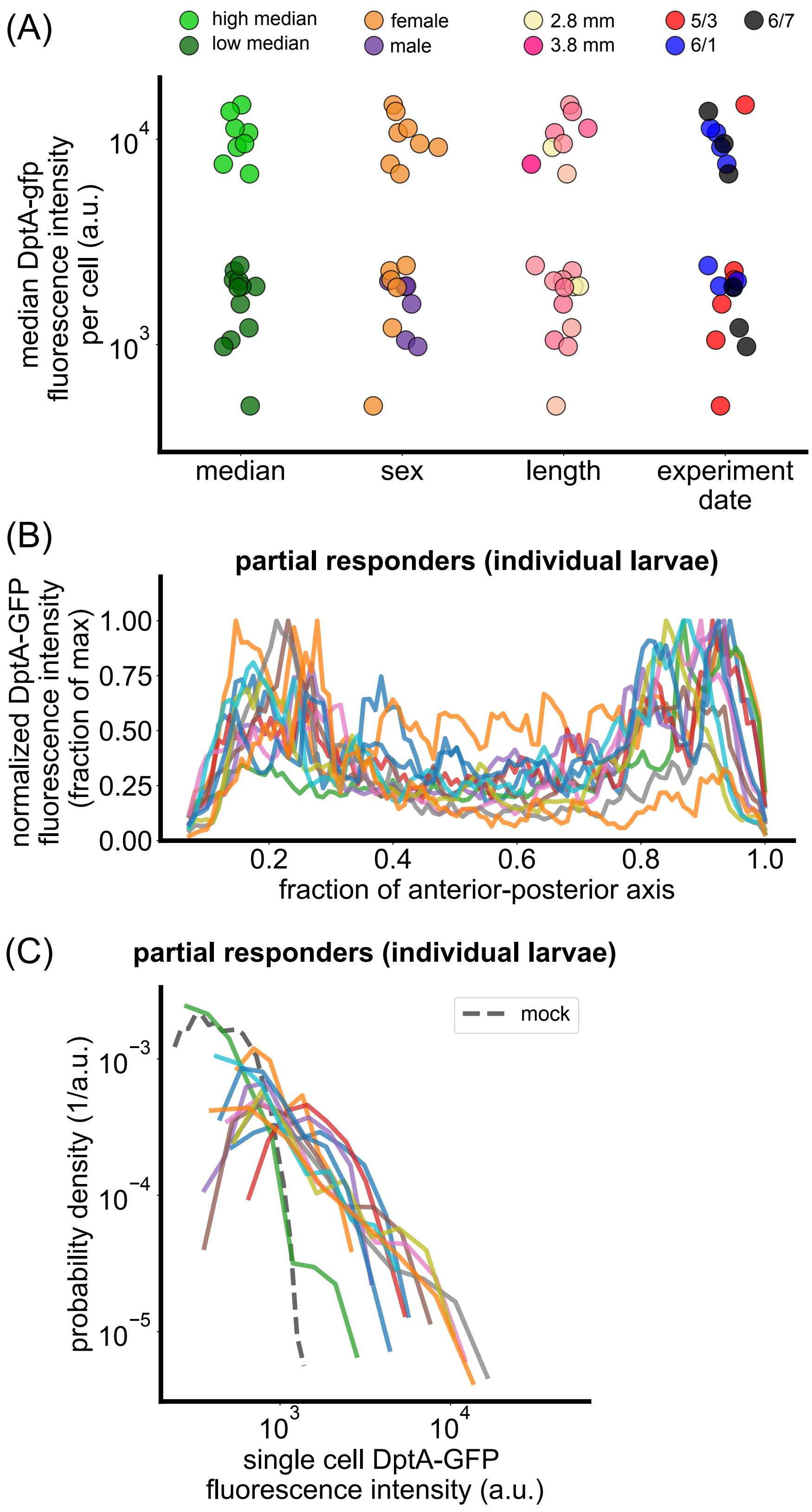

Figure S3: (Caption on next page)

bioRxiv preprint doi: https://doi.org/10.1101/2024.09.12.612587; this version posted September 16, 2024. The copyright holder for this preprint (which was not certified by peer review) is the author/funder, who has granted bioRxiv a license to display the preprint in perpetuity. It is made available under aCC-BY 4.0 International license.

Figure S3: (Previous page.) **Additional details of the partial DptA response** (A) Partial-complete split by different metadata. From left to right: original clustering by single-cell median DptA-GFP fluorescence intensity produces a clean separation; larva sex, where we see a partial correlation in that all the males observed are partial responders; length of the fat body, which is a proxy for developmental stage and thus Ecdysone levels, though we see no correlation; experiment date, to control for effects related to the details of experiment preparation and injections, where we see no correlation. (B) Normalized DptA-GFP distributions along the anterior posterior axis shown for each larva in the partial responses group. (C) Probability densities of single-cell DptA-GFP fluorescence intensity for each larva in the partial responses group.

bioRxiv preprint doi: https://doi.org/10.1101/2024.09.12.612587; this version posted September 16, 2024. The copyright holder for this preprint (which was not certified by peer review) is the author/funder, who has granted bioRxiv a license to display the preprint in perpetuity. It is made available under aCC-BY 4.0 International license.

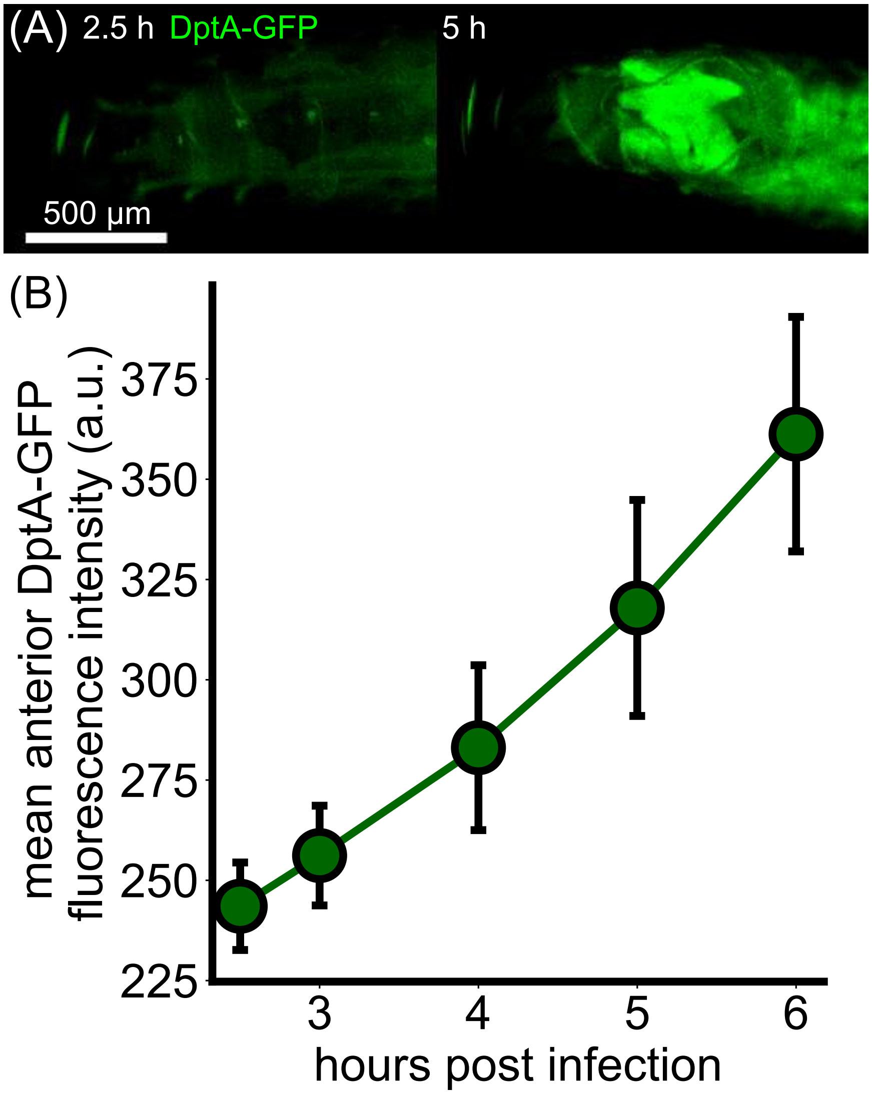

Figure S4: **Dynamics of DptA activation on a conventional widefield microscope mirror findings using light sheet fluorescence microscopy.** (A) Single z-plane images of the anterior of a larva carrying the DptA-GFP reporter taken at 2.5 hours post injection (left) and 5 hours post injection (right). (B) Quantification of DptA-GFP fluorescence intensity in the anterior fat body over time (mean and standard deviation over $N = 11$ larvae).

bioRxiv preprint doi: https://doi.org/10.1101/2024.09.12.612587; this version posted September 16, 2024. The copyright holder for this preprint (which was not certified by peer review) is the author/funder, who has granted bioRxiv a license to display the preprint in perpetuity. It is made available under aCC-BY 4.0 International license.

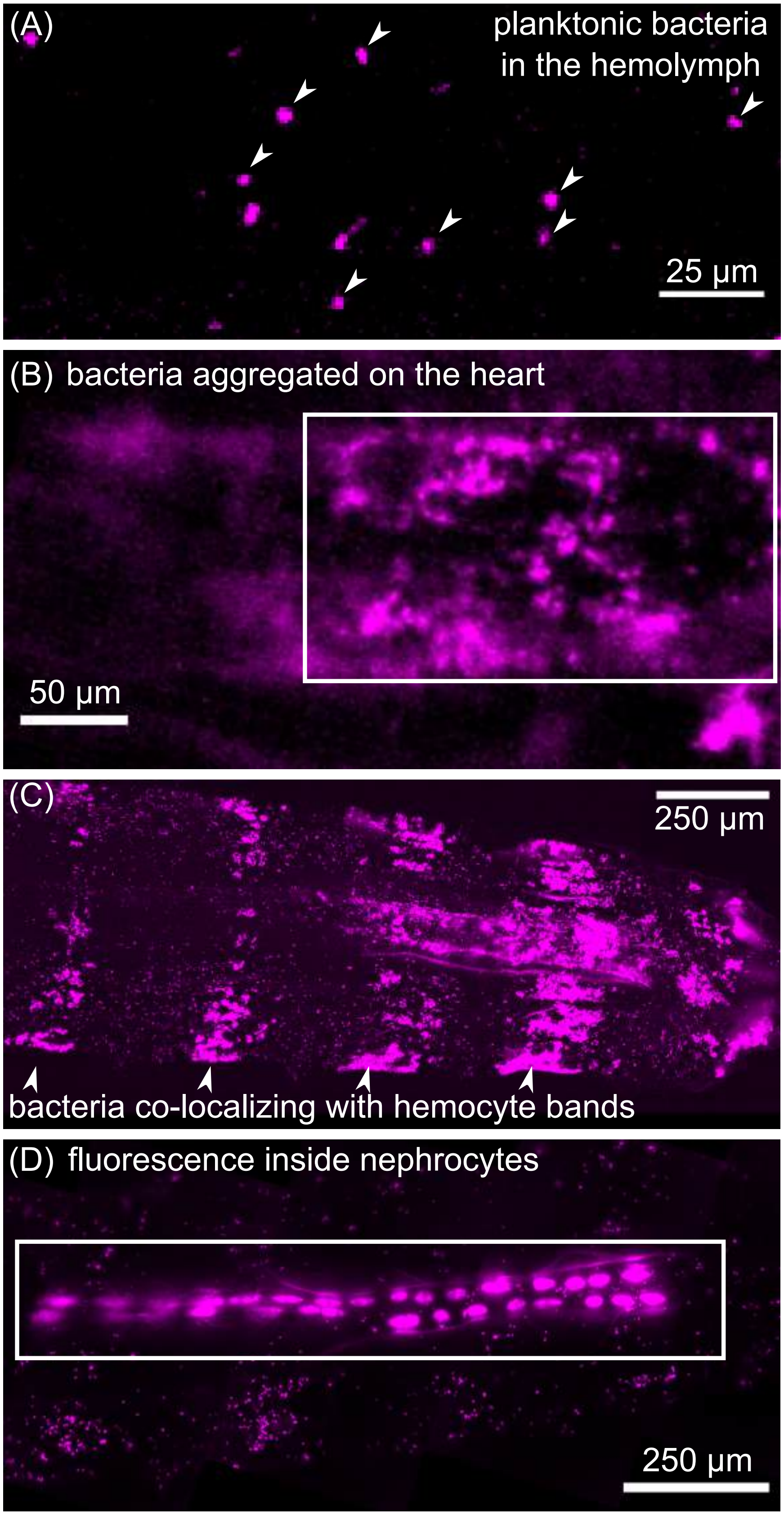

Figure S5: (Caption on next page)

bioRxiv preprint doi: https://doi.org/10.1101/2024.09.12.612587; this version posted September 16, 2024. The copyright holder for this preprint (which was not certified by peer review) is the author/funder, who has granted bioRxiv a license to display the preprint in perpetuity. It is made available under aCC-BY 4.0 International license.

Figure S5: **Gallery of bacterial localization.** (A) Single z-plane image planktonic bacteria in the hemolymph (arrow heads). These cells were identified as suspended freely in the hemolymph by their motion in subsequent z-planes. (B) Single z-plane image showing bacteria on the posterior end of the heart. (C) Maximum intensity projection image showing an example of bacteria co-localizing with known patterns of sessile hemocyte bands (arrow heads) [43]. (D) Maximum intensity projection image showing an example of bacteria internalized by nephrocytes embeded in the heart. Images in panels (A)-(C) are from 3-5 hours post injection. Panel (D) is from 6-8 hours post injection.

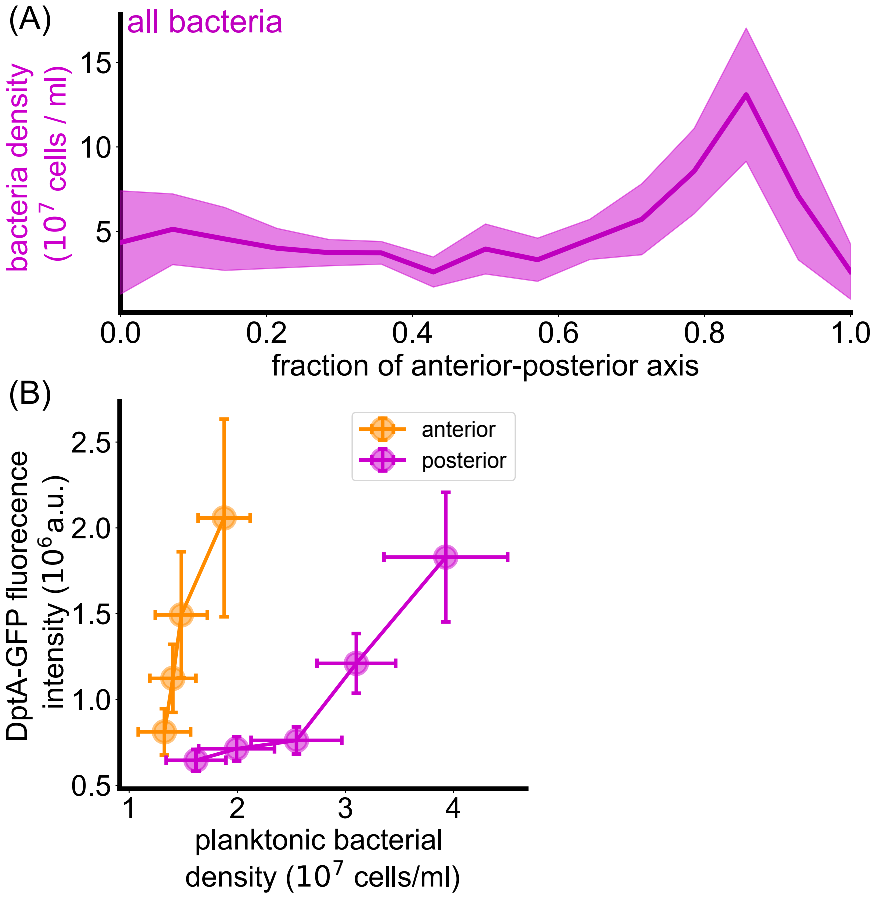

Figure S6: **Additional details of bacteria spatial distribution quantification**. (A) Spatial distribution of all bacteria. (B) Input-output functions for DptA-GFP vs. planktonic bacterial density for the anterior (orange) and posterior (magenta) fat body. The two regions were defined from the peak of DptA-GFP expression to the middle of the fat body, with one anterior-posterior axis bin separating the regions.

bioRxiv preprint doi: https://doi.org/10.1101/2024.09.12.612587; this version posted September 16, 2024. The copyright holder for this preprint (which was not certified by peer review) is the author/funder, who has granted bioRxiv a license to display the preprint in perpetuity. It is made available under aCC-BY 4.0 International license.

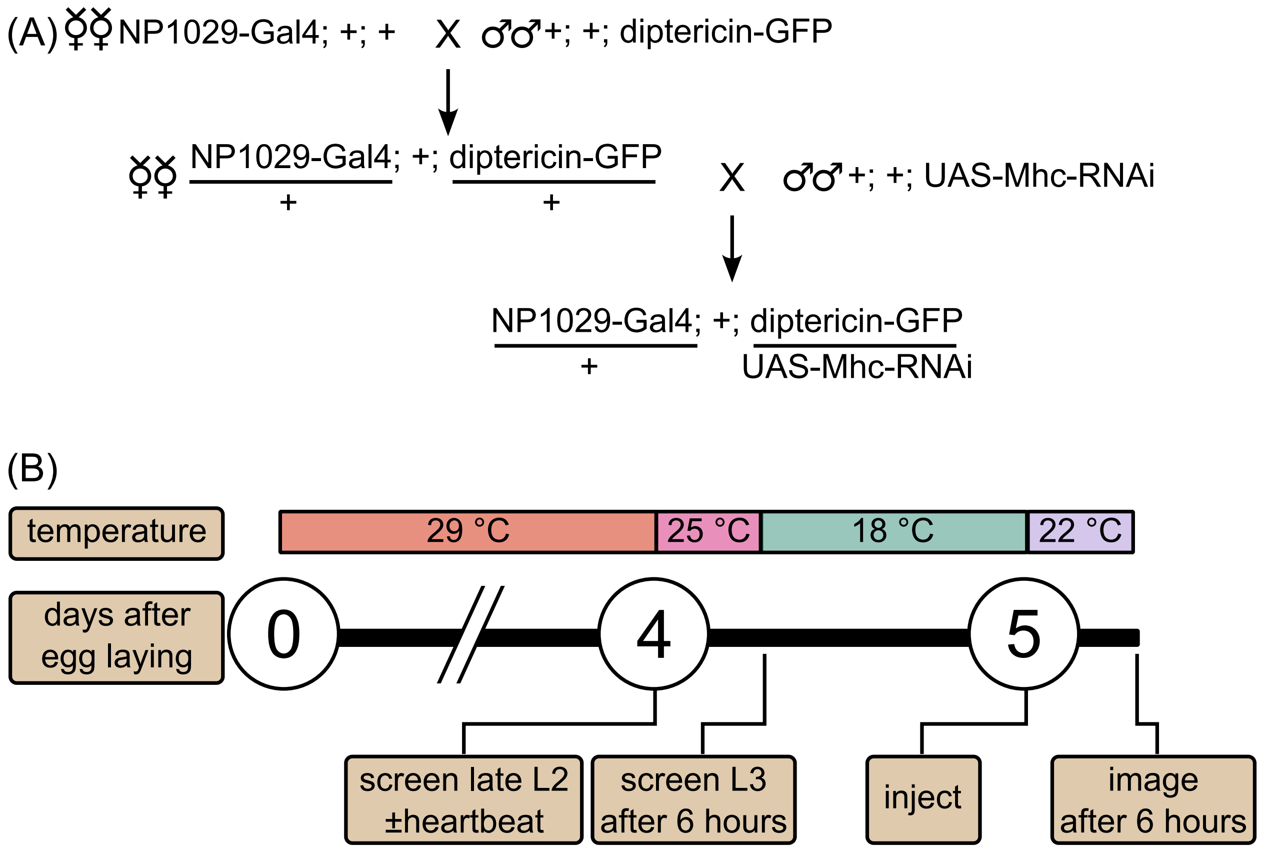

Figure S7: **Details of the heartbeat knockdown experiment** (A) Fly crossing scheme for generating flies lacking a heartbeat and containing the DptA reporter. (B) Schematic of the timeline and temperatures used in the heartbeat knockdown experiment.

bioRxiv preprint doi: https://doi.org/10.1101/2024.09.12.612587; this version posted September 16, 2024. The copyright holder for this preprint (which was not certified by peer review) is the author/funder, who has granted bioRxiv a license to display the preprint in perpetuity. It is made available under aCC-BY 4.0 International license.

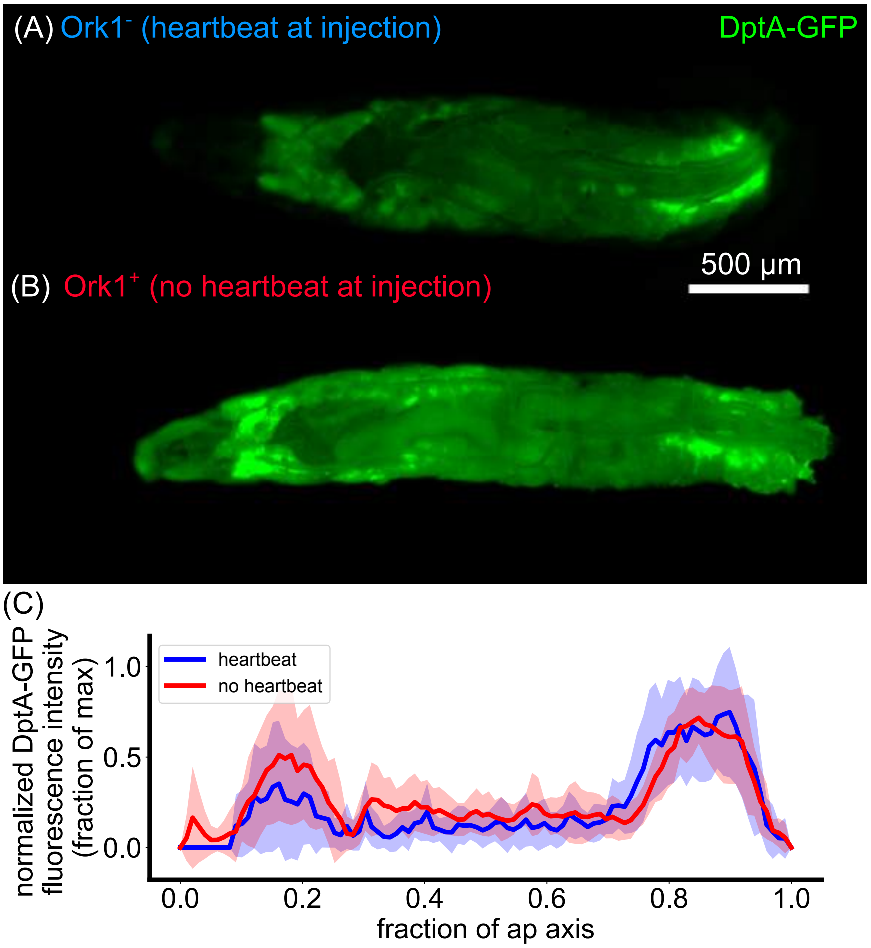

Figure S8: **Temporary loss of heartbeat by overexpression of Ork1 results in no change in the spatial pattern of DptA-GFP expression.** (A)-(B) Single z-plane widefield images of larvae 24 hours after infection for larvae that either had (A) or did not have (B) a heartbeat at the time of injection. Loss of heartbeat was achieved via the larval heart-specific driver NP1029>Ork1 (the crossing scheme was identical to the scheme in Fig. S7A). Starting approximately 3 hours after injection, heartbeats begin to beat again (Supplemental Movie 9). (C) Quantification of DptA-GFP spatial pattern (mean and standard deviation across $N = 6$ larvae for heartbeat$^+$ group, $N = 8$ for heartbeat$^-$ group) normalized to the maximum value for each larva across anterior-posterior bins.

bioRxiv preprint doi: https://doi.org/10.1101/2024.09.12.612587; this version posted September 16, 2024. The copyright holder for this preprint (which was not certified by peer review) is the author/funder, who has granted bioRxiv a license to display the preprint in perpetuity. It is made available under aCC-BY 4.0 International license.

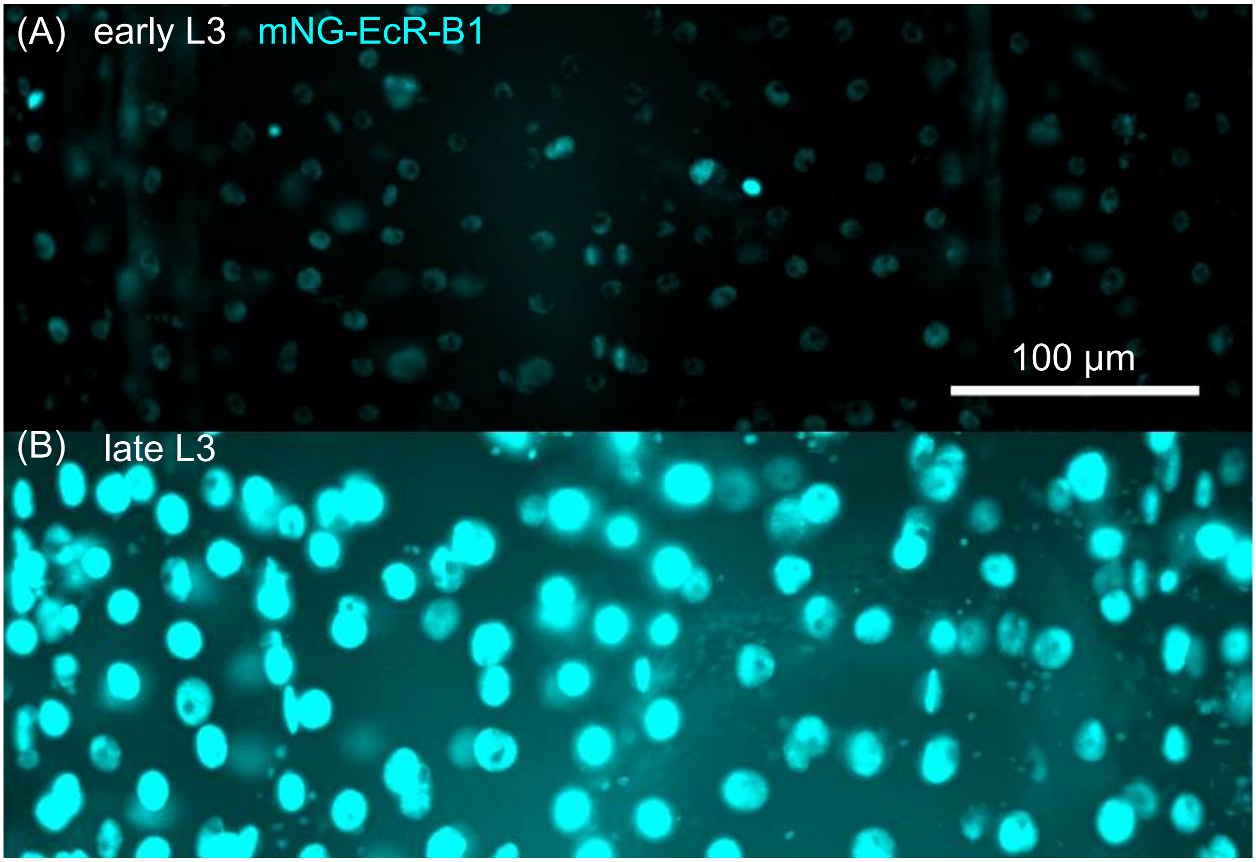

Figure S9: **Nuclear mNeonGreen-EcR-B1 fluorescence intensity correlates with developmental stage** (A)-(B) Maximum intensity projections of mNeonGreen-EcR-B1 images in early (A) and late (B) third instar larvae. Image regions correspond to areas approximately above the anterior-dorsal lobes of the fat body, but the images are not masked by fat body nuclei, so contain signal from multiple cell types.

bioRxiv preprint doi: https://doi.org/10.1101/2024.09.12.612587; this version posted September 16, 2024. The copyright holder for this preprint (which was not certified by peer review) is the author/funder, who has granted bioRxiv a license to display the preprint in perpetuity. It is made available under aCC-BY 4.0 International license.

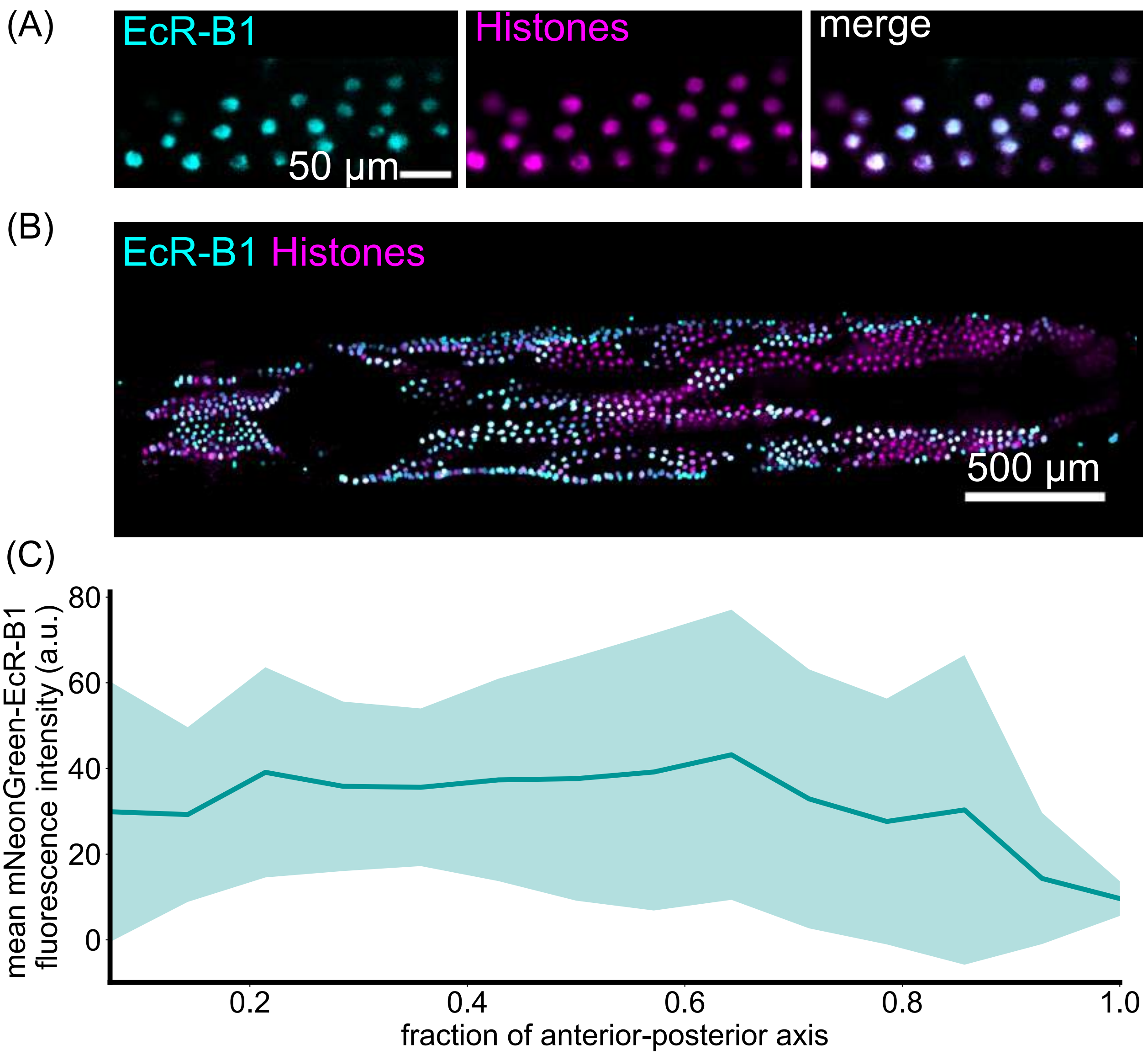

Figure S10: **Nuclear localization of Ecdysone receptor (EcR), a transcriptional regulator of Diptericin, does not correlate spatially with DptA-GFP expression.** (A) An example single z-slice of middle-dorsal fat body nuclei showing raw fluorescence of mNG-EcR-B1 (left, cyan), fat body histones marked by cg-Gal4 x UAS-HisRFP (middle, magenta), and the merged image. (B) Maximum intensity projection of a full view of the larval fat body showing mNG-EcR-B1 localization. While the localization pattern exhibits some degree of local structure, unlike DptA-GFP, it is broadly uniform along the anterior-posterior axis. Fat body nuclei were computationally segmented and then false colored (cyan channel) by their mean background-subtracted mNG-EcR-B1 fluorescence intensity (Methods). In this way, EcR levels in non-fat body cells are not visualized. Note that cg-Gal4 also labels hemocytes, but hemocytes are computationally removed based on their smaller size (Methods). (C) Quantification of nuclear-localized mNG-EcR-B1 levels along the anterior-posterior axis. Solid line and shaded error bars are the mean and standard deviation respectively across $N = 6$ larvae.

bioRxiv preprint doi: https://doi.org/10.1101/2024.09.12.612587; this version posted September 16, 2024. The copyright holder for this preprint (which was not certified by peer review) is the author/funder, who has granted bioRxiv a license to display the preprint in perpetuity. It is made available under aCC-BY 4.0 International license.

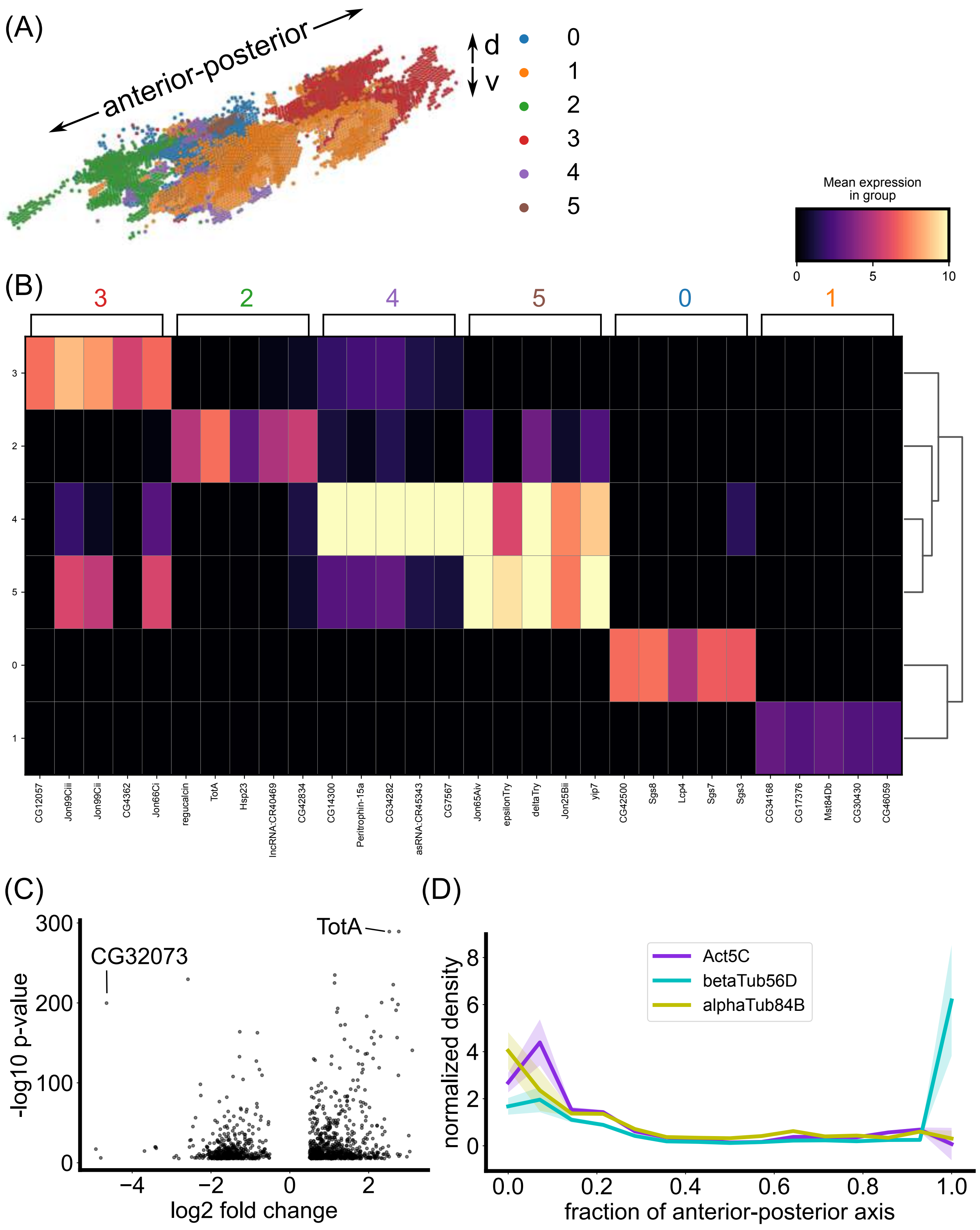

Figure S11: (Caption on next page)

bioRxiv preprint doi: https://doi.org/10.1101/2024.09.12.612587; this version posted September 16, 2024. The copyright holder for this preprint (which was not certified by peer review) is the author/funder, who has granted bioRxiv a license to display the preprint in perpetuity. It is made available under aCC-BY 4.0 International license.

Figure S11: **Additional details of spatial transcriptomics analysis** (A) 3D rendering of fat body cells colored and labelled by Leiden cluster. (B) Matrix plot showing the top 5 marker genes defining each cluster. (C) Volcano plot showing results of differential expression analysis between the anterior fat body region, cluster 2, and the rest of the tissue. (D) Examples of anterior patterning in housekeeping genes. The anterior peak of these patterns is further anterior from the peak of antimicrobial expression, which occurs between 0.15 and 0.2 anterior-posterior fraction.

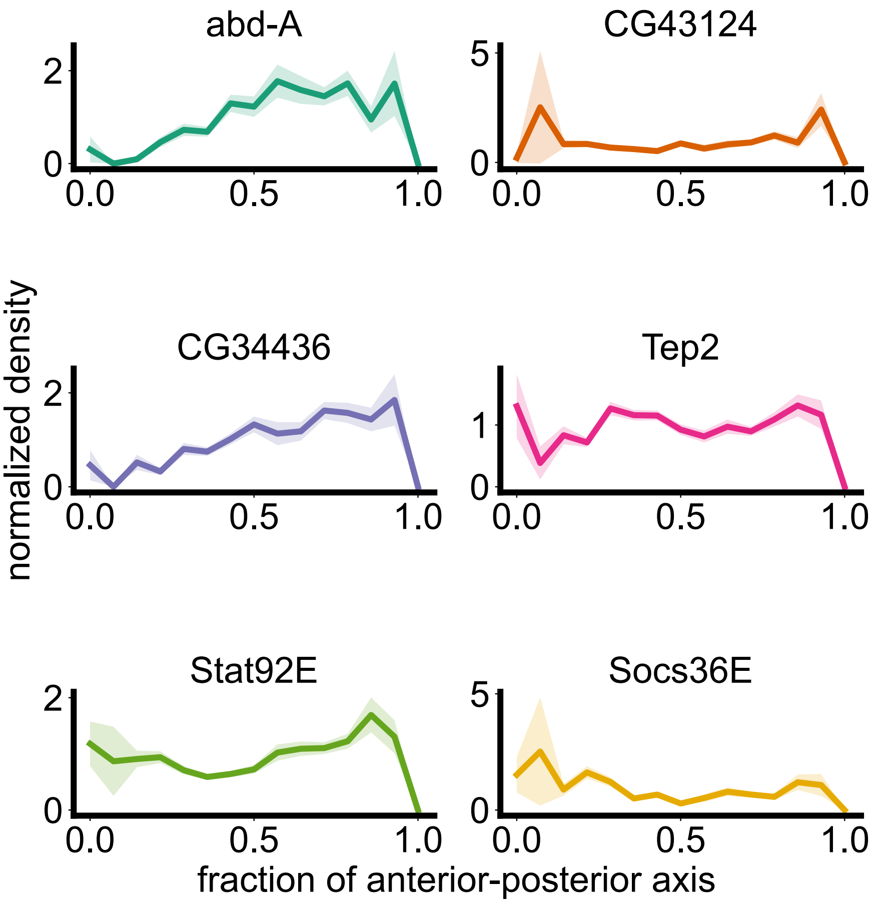

Figure S12: **Spatial transcriptomics data recapitulates known genes with posterior peaks**. These genes were identified in a differential expression analysis of bulk RNA seq from dissected tissue fragments and being enriched in the posterior compared to the middle-lateral fat body [48]. Expression patterns are normalized to integrate to unity. The first gene, abd-A, is a Hox gene involved in anterior-posterior patterning. Some genes also exhibit a peak in the anterior fat body, which was not included in [48].
